# 3D assessment of intervertebral disc degeneration in zebrafish identifies changes in bone density that prime disc disease

**DOI:** 10.1038/s41413-021-00156-y

**Published:** 2021-08-31

**Authors:** Erika Kague, Francesco Turci, Elis Newman, Yushi Yang, Kate Robson Brown, Mona S. Aglan, Ghada A. Otaify, Samia A. Temtamy, Victor L. Ruiz-Perez, Stephen Cross, C. Patrick Royall, P. Eckhard Witten, Chrissy L. Hammond

**Affiliations:** 1 School of Physiology, Pharmacology and Neuroscience, Biomedical Sciences, University of Bristol, Bristol, UK; 2 School of Physics, HH Wills Physics Laboratory, University of Bristol, Bristol, UK; 3 Centre for Nanoscience and Quantum Information, University of Bristol, Bristol, UK; 4 Bristol Centre for Functional Nanomaterials, University of Bristol, Bristol, UK; 5 Department of Anthropology and Archaeology, University of Bristol, Bristol, UK; 6 Department of Mechanical Engineering, University of Bristol, Bristol, UK; 7 Clinical Genetics Department, Human Genetics and Genome Research Division, Center of Excellence for Human Genetics, National Research Centre, Cairo, Egypt; 8 Instituto de Investigaciones, Biomedicas de Madrid, and Ciber de Enfermedades Raras (CIBERER), Madrid, Spain; 9 Wolfson Bioimaging Facility, Biomedical Sciences, University of Bristol, Bristol, UK; 10 School of Chemistry, University of Bristol, Bristol, UK; 11 Evolutionary Developmental Biology, Department of Biology, Ghent University, Ghent, Belgium

**Keywords:** Bone, Bone quality and biomechanics, Pathogenesis

## Abstract

Back pain is a common condition with a high social impact and represents a global health burden. Intervertebral disc disease (IVDD) is one of the major causes of back pain; no therapeutics are currently available to reverse this disease. The impact of bone mineral density (BMD) on IVDD has been controversial, with some studies suggesting osteoporosis as causative for IVDD and others suggesting it as protective for IVDD. Functional studies to evaluate the influence of genetic components of BMD in IVDD could highlight opportunities for drug development and repurposing. By taking a holistic 3D approach, we established an aging zebrafish model for spontaneous IVDD. Increased BMD in aging, detected by automated computational analysis, is caused by bone deformities at the endplates. However, aged zebrafish spines showed changes in bone morphology, microstructure, mineral heterogeneity, and increased fragility that resembled osteoporosis. Elements of the discs recapitulated IVDD symptoms found in humans: the intervertebral ligament (equivalent to the annulus fibrosus) showed disorganized collagen fibers and herniation, while the disc center (nucleus pulposus equivalent) showed dehydration and cellular abnormalities. We manipulated BMD in young zebrafish by mutating *sp7* and *cathepsin K*, leading to low and high BMD, respectively. Remarkably, we detected IVDD in both groups, demonstrating that low BMD does not protect against IVDD, and we found a strong correlation between high BMD and IVDD. Deep learning was applied to high-resolution synchrotron µCT image data to analyze osteocyte 3D lacunar distribution and morphology, revealing a role of *sp7* in controlling the osteocyte lacunar 3D profile. Our findings suggest potential avenues through which bone quality can be targeted to identify beneficial therapeutics for IVDD.

## Introduction

Back pain is a global healthcare concern and economic burden. It is the leading cause of years lived with disability, with estimates that over 80% of adults will suffer from symptoms at some time in their lives^1^. Intervertebral disc degeneration (IVDD) is one of the major causes of back pain symptoms, one of the first pathological signs, and a potential target for intervention^2^. As the global population ages, a substantial increase in morbidity due to degenerative diseases and back pain is expected;^3^ this increase emphasizes the importance of improving our understanding of the causes of IVDD and its relationship with other common degenerative conditions to revise or further develop novel therapeutic strategies.

Intervertebral discs (IVDs) connect consecutive vertebral bodies; their main function is mechanical, and they act as shock-absorbing cushions^4^. Mammalian discs have an exquisite three-dimensional (3D) architecture comprised of the outer annulus fibrosus (AF) made from fibrocartilage and an inner region, the nucleus pulposus (NP). The NP is derived from the notochord; it contains chondrocyte-like cells and a gelatinous matrix composed of collagen type 2 and proteoglycans that are vital for the function of the discs and serve as a fluid-filled shock-absorbing cushion. IVDs are avascular; nutrients are delivered through diffusion at the endplates and AF^5,6^. While fissures and bulging AF are commonly found during aging, progressive loss of proteoglycans, water, and nutrient content leads to irreversible cellular and mechanical changes in the NP^2^. Aging, spine deformities, injuries, diseases, and genetic factors are involved in the pathogenesis. Several studies have highlighted some of the genetics of IVDD^7^, which include genes involved in bone and cartilage homeostasis (i.e., collagens, metalloproteinases, vitamin D, *GDF5,* and *IL-6*)^7–9^. While genome-wide association studies (GWAS) have identified an association of a few loci with back pain, IVDD endophenotypes, larger samples, replication, and functional studies are needed to improve reliability and to identify genes associated specifically with IVDD^7,8,10,11^.

The association between IVDD and bone mineral density (BMD) has been controversial. It has long been proposed that reduced bone quality leads to progressive endplate degradation and spondylosis, ultimately culminating in increased disc degeneration^12^. However, patients with low BMD, despite having higher risks of vertebral body fractures, seem to show reduced rates of IVDD^13–15^. According to this hypothesis, IVDD would be delayed by osteoporosis, an aging-related degenerative and debilitating condition that affects millions of people worldwide^16^. However, the long-term effects of osteoporosis or poor bone quality are far from resolved, as other studies have reported a positive association with disc degeneration^17–19^, and treatments used for osteoporosis, such as alendronate or calcitonin retarded IVDD in ovariectomized rats^18,20–22^. On the other hand, a positive association of higher lumbar spine BMD with IVDD has been suggested from cadaveric studies^23^, a UK Twin Study, and populational studies^24,25^. Functional studies to understand the relationship between the genetics of BMD and IVDD integrity could clarify this relationship and aid in the identification of new therapeutic opportunities.

Zebrafish (*Danio rerio*) provide an attractive teleost model to study adult skeletal conditions, including those related to osteoporosis, osteoarthritis, and spinal deformities^26,27^. Zebrafish carrying mutations in genes associated with osteoporosis and osteogenesis imperfecta exhibit frequent fractures in the ribs and low BMD^28–31^. In teleosts, the connections of the endplates of vertebral bodies are similar in design and function to mammalian IVD (hereafter called IVD). Centrally located vacuolated notochord cells with the same function as NP (hereafter called the NP) are surrounded by a strong composite ligament that functions like the AF (hereafter called the AF)^32^. The ligament is composed of, from inside to outside, collagen type II embedded in a matrix resembling cartilage, elastin, and collagen type 1 fiber bundles^33^. In adult teleosts, vacuolated notochord cells turn into fibrous, keratinized, connective tissue (the septum and notochord strand) and extracellular vacuoles^32,34^. Disc damage and subsequent vertebral body fusion have been reported in farmed Atlantic salmon, suggesting that zebrafish could be a potential degenerative model for IVD^35^. We have shown frequent spinal deformities in aged zebrafish, including spinal curvatures, osteophytosis, and changes akin to those of osteoarthritis^36^. Recently, Monma et al. showed reduced trabecular volume and number, similar to the reductions observed in aging humans^37^. Moreover, genetic zebrafish models displaying BMD fluctuations are attractive tools for functional evaluation of the interplay between BMD and IVDD.

Here, we report for the first time IVDD in adult zebrafish. We describe microstructure alterations of the vertebral bone, showing local changes in bone mineral distribution, the osteocyte lacunar profile, and collagen fiber organization, demonstrating that deterioration of bone quality in aged fish leads to increased fragility, as is seen in individuals with osteoporosis. In the IVD, we characterized changes to bone and soft tissue involving collagen fibers of the AF, dehydration, fibrosis (scarring tissue), and cellular changes in the NP. To address the controversy over the relationship of BMD with IVDD, we analyzed the spines from zebrafish with low BMD (*sp7*^*−/−*^) and high BMD by genetically manipulating *cathepsin K*. Interestingly, both zebrafish mutant lines showed premature IVDD, contradicting the idea that low BMD is a protective factor in IVDD. A positive correlation between low BMD and the incidence of IVDD and between high BMD and the incidence of IVDD was observed, suggesting a U-shaped model in which deviation in either direction from normal BMD increases the risk of developing IVDD. By analyzing changes in bone quality that precede the detection of BMD fluctuations, we suggest that therapeutics targeting improving bone quality can potentially help in the prevention and treatment of disc disease.

## Results

### Radiographic signs of IVDD in the aging zebrafish vertebral column

**Fig. 1:**
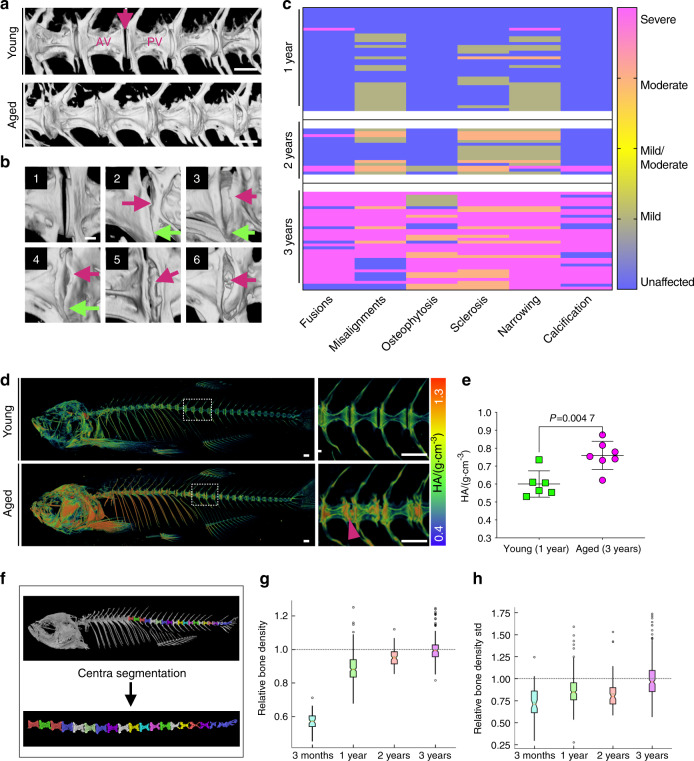
Progressive abnormalities found in aged zebrafish vertebral columns. **a** 3D rendering from μCT images of young (1 year) and aged (3 years) spines. AV anterior vertebra, PV posterior vertebra, IVD (arrow). Scale bar = 500 μm. **b** Frequent changes observed at the IVD. 1: normal IVD; 2: osteophytes (pink arrow), vertebral misalignment and IVD narrowing (green arrow); 3: endplate sclerosis; 4: sclerosis (pink arrow) and IVD narrowing (green arrow); 5: sclerosis and fusion (pink arrow); 6: sclerosis and IVD calcification (pink arrow). Scale bar = 100 μm. **c** Heat map graph showing spinal morphological changes classified by severity during aging (1 year *n* = 36 (42% females, 58% males), 2 years *n* = 16 (57% females, 43% males), and 3 years *n* = 34 (55% females, 45% males)). Average fish standard lengths (measured from tip of the head to the last vertebral column): 1 year = 3.22 cm (0.19 SD), 2 years = 3.41 cm (0.18 SD), and 3 years = 3.46 cm (0.23 SD). **d** 3D rendering from μCT images of young and aged fish, color coded to show bone mineral density changes. The selected area of the spine (dashed box) is magnified, as shown on the right of the panel. Higher density colocalizes with regions of sclerosis and deformities (arrowhead) in the aged spine. Scale bar = 500 μm. **e** TMD (tissue mineral density) retrieved from the third thoracic vertebrae in young (1 year) and aged (3 years) fish. Nonparametric, two-tailed, Mann–Whitney test; data are the mean and SD. *P* values are indicated. **f** 3D volume rendering from a μCT image of wt fish showing an individual vertebral centrum segmented by computational automation. **g** Relative bone density from the vertebral centra in aging fish (3 months to 3 years). Average standard lengths: 3 months = 2.6 cm (0.18 SD); 3 years = 3.46 cm (0.23 SD). The notch plot was scaled by the average value from the 3-year centra. **h** Within-sample standard deviation in bone density. The notch plot was scaled by its average value from the 3-year centra. **c**, **e** Generated in Prism 8. **g**, **h** Graph was generated in Python

Radiographic signs of IVDD in humans typically include osteophytosis, endplate sclerosis, and disc space narrowing^38^. To identify progressive changes in the spine during aging that could act as markers for IVDD, we analyzed microcomputed tomography (μCT) image data of 1- (*n* = 36), 2- (*n* = 16) and 3- (*n* = 34) year-(y)-old wild-type (wt) zebrafish, followed by spinal abnormality classification based on severity (Fig. 1a, b). With increasing age, zebrafish exhibited increased severity of vertebral fusion, misalignments, osteophytes, endplate sclerosis, uneven disc spacing, IVD narrowing and disc calcification (Fig. 1b, c). Fifty-seven percent of all fusions and disc narrowing occurrences were found between vertebrae in the rib-bearing abdominal region. Degenerative changes in the vertebral centra can be detected by simple radiographs or using Alizarin Red S staining (Supplementary Fig. 1). To understand whether vertebral bodies display shape changes during aging, we performed two-dimensional (2D) morphometric analysis on the third transitional vertebra from a subset of young and aged fish (*n* = 8) using lateral μCT images. Shape variation confirmed modification during aging (*P* = 0.043). Interestingly, the highest deformation (standard deviation from the normal shape) corresponded to sclerotic endplates (Supplementary Fig. 2). Thus, aging zebrafish develop radiographic signs of IVDD.

### Elevated bone density and increased risk of fractures in aged zebrafish spines: are the bones of aged zebrafish osteoporotic?

**Fig. 2:**
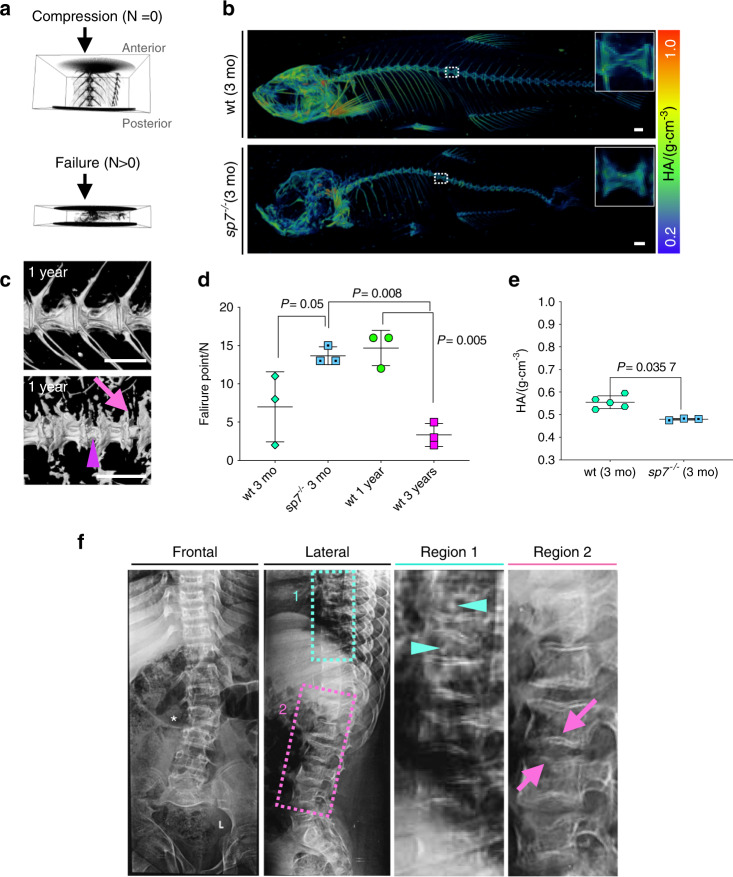
Altered vertebral column biomechanics in aged and *sp7*^−/−^ zebrafish show increased bone fragility. **a** Intact vertebral column motion segments (composed of three vertebrae surrounded by trunk musculature) were placed in a material testing stage (MTS), followed by μCT imaging at increasing compression forces. An example of vertebral column segments before compression (N = 0) and after failure (N > 0) is shown. Anterior and posterior orientations of the zebrafish are annotated. **b** 3D volumetric rendering from μCT images of 3-month-old wt and *sp7*^*−/−*^ zebrafish color coded for tissue mineral density (TMD). The dashed box shows the magnified region presented on the right of the panel. Scale bars = 500 μm. **c** Volumetric rendering from μCT images of 1-year-old wt and *sp7*^*−/−*^ zebrafish. Note IVD calcification (arrowhead) and abnormal shapes of the centra (arrow) in *sp7*^*−/−*^ zebrafish. Scale bars = 500 μm. **d** Failure points during compressive forces of 3-month-old (3 mo) *sp7*^*−/−*^ zebrafish, wt zebrafish siblings, 1-year-old wt siblings, and 3-year-old wt siblings (*n* = 3 per group). One-way ANOVA and post hoc Holm–Sidak’s multiple comparisons test were used; data are the mean and SD. *P* values are indicated. The graph was generated in Prism 8. Scale bars = 500 μm. **e** TMD of 3-month-old (3 mo) wt and s*p7*^*−/−*^ zebrafish (wt *n* = 5 (3 males +2 females), *sp7*^*−/−*^ zebrafish *n* = 3 (2 males + 1 female)). Standard fish lengths: wt = 2.43 cm (0.2 SD); *sp7*^*−/−*^ = 2.17 (0.4 SD). Nonparametric, two-tailed, Mann–Whitney test; data are the mean and SD. *P* values are indicated. **f** Radiograph of a 16-year-old male with a frameshift mutation in *SP7* (c.1052delA). Frontal and lateral images are displayed. Regions 1 and 2 are magnified and displayed on the right. Lumbar spinal curvature (asterisk), biconcave vertebrae (pink arrows), the spread of IVD, and signs of IVD calcification (cyan arrowhead) are shown

To study BMD and IVDD using zebrafish, we analyzed μCT image data with grayscale calibration for BMD, comparing 3D volumetric renders of young (1 year) and aged (3 years) zebrafish. Regions of higher density corresponded to the greatest morphometric variations at endplates, while lower density was observed in the middle of the centra (Fig. 1d). Increased centra tissue mineral density (TMD) was detected when the same centrum was compared among spines (*P* = 0.004 7) (Fig. 1e). We analyzed a higher number of centra in aging zebrafish by automated centra segmentation (Fig. 1f and Supplementary Fig. 3). Due to the complex morphology of the abdominal region leading to difficulties in separating the centra from the ribs and the highest mechanical load of the caudal vertebral bodies^39^ (similar to the human lumbar spine), we excluded those centra in the abdominal region from our analysis. We analyzed 644 vertebral centra from 3-month, 1-, 2- and 3-year-old zebrafish (63, 291, 83, and 207 centra, respectively). Although the number of vertebral centra analyzed was different between the groups, potentially causing statistical bias in the automated analysis, we observed a gradual increase in bone density from 3-month-old to 3-year-old samples (Fig. 1g), among other measurements retrieved (Supplementary Fig. 4). The heterogeneous distribution of bone tissue minerals is an indicator of poor bone quality and fracture risk^40,41^. We hypothesized that uneven BMD would alter the material and structural properties of aged bone and result in increased fragility. The standard deviation within samples suggested an increased mineral heterogeneity distribution in aged spines (Fig. 1i). To test the performance of the vertebral column, we applied compressive force to a section of the spine (anterior to posterior axis) ex vivo using a material testing stage (MTS) within a μCT machine; this approach allowed us to monitor the point of failure of the vertebral column in a protocol previously described^42^. We tested young (1 y) and aged (3 y) samples (*n* = 3 for each group). While young spines failed at 15 N, aged spines failed at 3.33 N (*P* = 0.005) (Fig. 2a, d and Supplementary Fig. 5G). The results demonstrate that a focal increase in bone density colocalizes with deformed endplates in aging zebrafish; uneven mineral density distribution supports poor bone quality, leading to increased fragility. Our results corroborate human populational studies demonstrating that degenerative changes of the lumbar spine may lead to falsely elevated BMD values and cause underdiagnosis of osteoporosis^43^. Despite increased bone density at zebrafish endplates, the bones of aged zebrafish are osteoporotic.

### Young zebrafish with low BMD show premature radiographic IVDD

The study of aging zebrafish to disentangle the association between BMD and IVDD is complex, as our data indicate increased BMD associated with IVDD in aging fish; furthermore, increased fragility in aged bones, resembling osteoporosis, was also associated with IVDD in aging zebrafish. To probe this relationship, we analyzed the vertebral column and IVD of young fish with genotypes associated with reduced bone density. *Sp7* is a major osteogenesis transcription factor expressed in osteoblasts^44,45^. Genome-wide association studies (GWAS) have identified associations between *SP7* and BMD^46^, between *SP7* and lumbar spine BMD and between *SP7* and estimated BMD (musculoskeletal knowledge portal, mskkp.org). The *SP7* locus was also identified in a meta-analysis evaluating gene variants associated with BMD across the lifespan^47^. Mutations in *SP7* cause recessive osteogenesis imperfecta (OI type XII), characterized by generalized osteopenia, recurrent fractures, bone deformities and bent bones^48–50^. Zebrafish *sp7*^*−/−*^ show frequent fractures in the ribs, skull abnormalities (Wormian bones) and tooth phenotype, similar to those found in human patients^31,51^. By performing μCT image analysis of young (3 months, mo) *sp7*^*−/−*^ zebrafish, we observed a dramatic reduction in TMD in comparison to wt siblings (*p* = 0.034) (Fig. 2b, e and Supplementary Fig. 5E). Interestingly, endplate sclerosis, calcification and broadening of the IVD (Fig. 2b, c and Supplementary Fig. 5D) suggest premature bone degenerative changes. The compressive loading experiment for the section of *sp7*^*−/−*^ spine (3 mo) and comparative sibling samples showed that *sp7*^*−/−*^ areas bent more and suddenly failed at 14.67 N (*P* = 0.05) (Fig. 2d and Supplementary Fig. 5F, G). Bent long bones have been described in a human patient homozygous for a recessive mutation in *SP7* (c.1052delA, p.(Glu351Glyfs*19))^48^, while vertebral column curvature has also been reported^49,50^, IVD changes have not been described to date. We contacted the Egyptian child with the *SP7* mutation reported in 2010;^48^ now 15 years old, the patient displayed 2–3 fractures per year and was unable to walk or stand. Radiographs of the patient’s trunk showed mild spinal curvature (kyphoscoliosis), abnormal vertebral shape (biconcave wedging, “cod shape”), endplate sclerosis, more areas affected by IVD and signs of calcification (Fig. 2f and Supplementary Fig. 6), similar to our findings in zebrafish. Therefore, the *sp7*^*−/−*^ vertebral column recapitulates changes observed in a human *SP7* patient. Our results indicate an association of *SP7*, low BMD, and the incidence of IVDD.

### Mineral distribution heterogeneity, reduced mineralization robustness, and reduced bone quality in aged bones substantiate osteoporosis in aged zebrafish

**Fig. 3:**
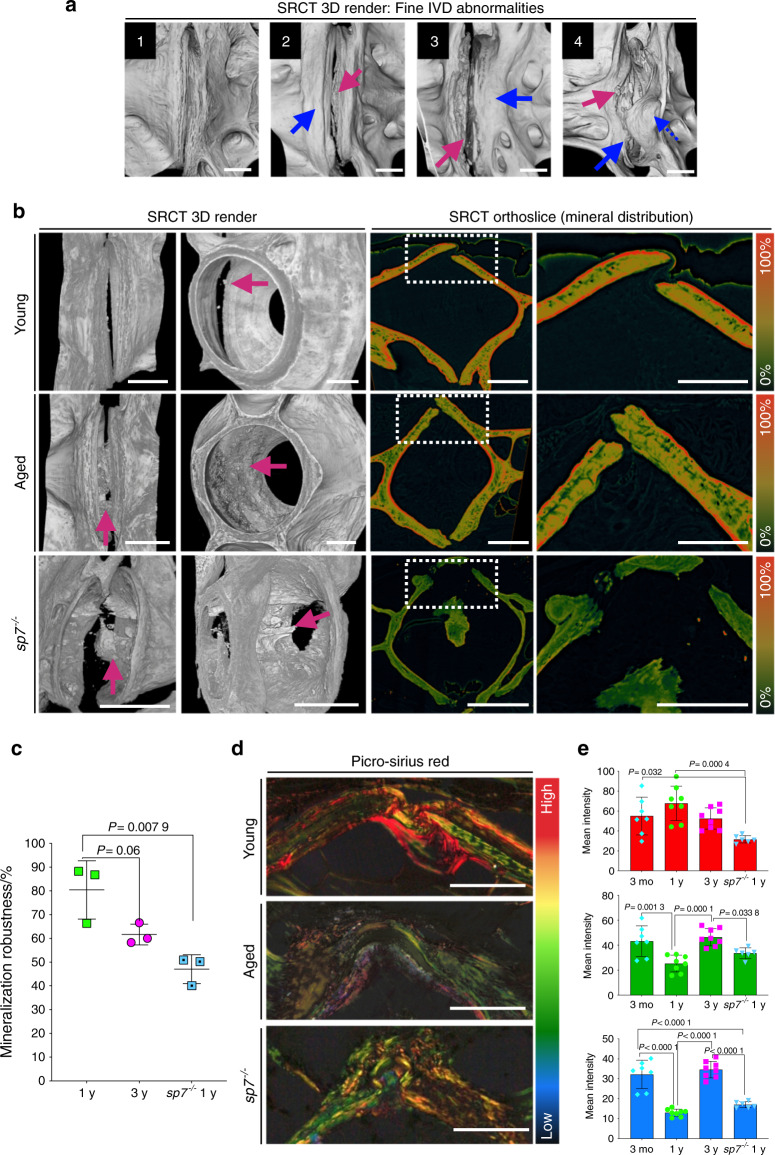
SRCT reveals subtle bone morphological abnormalities and alterations in mineral density distribution. **a** 3D volumetric rendering from μCT images showing fine endplate abnormalities. 1: normal IVD; 2: endplate sclerosis (blue arrow) and IVD calcification (pink arrow); 3: sclerosis (blue arrow), uneven edges and AF calcification (pink arrow); 4: sclerosis (blue arrow), AF calcification (pink arrow); osteophyte (dashed blue arrow). Scale bars = 50 μm. **b** 3D volumetric rendering from μCT images showing IVD internal changes in aged and *sp7*^*−/−*^ zebrafish. Calcification depicted by pink arrow (SRCT 3D rendering). Note, young zebrafish with IVD and disc misalignment shows points of IVD calcification. Virtual sections (orthoslices) color coded for bone density (SRCT orthoslice). Dashed box regions are magnified and displayed on the right. Scale bars = 50 μm. **c** Quantification of mineralization robustness (%) in wt young (1 y), wt aged (3 y), and *sp7*^*−/−*^ 1 y zebrafish (*n* = 3 per group, average from 4 sections per fish). One-way ANOVA and post hoc Holm–Sidak’s multiple comparisons test were used; data are the mean and SD. *P* values are indicated. **d** Paraffin sections stained with picrosirius red showing the thickness of collagen fibers (thin = blue/green and thick= red/orange). Scale bars = 50 μm. **e** Quantification of collagen fiber thickness from picrosirius red staining (blue = thin, green = medium, red = thick) (1 y, *n* = 8; 3 y, *n* = 8; 3 months, *n* = 7; *sp7*^*−/−*^ 1 y, *n* = 6; we analyzed three sections per fish). One-way ANOVA, post hoc Tukey’s multiple comparisons test; data are the mean and SD. *P* values are indicated

The biomechanical properties of bone are affected not only by the total amount of minerals but also by the distribution and organization of minerals within the nonmineralized matrix^33,52–54^. We imaged young (1 y), aged (3 y), and *sp7*^*−/−*^ (1 y) vertebrae using synchrotron radiation-based μCT (SRCT) (0.33–0.68 μm isotropic resolution, *n* = 3 fish per group) (Fig. 3a), allowing observation of subtle 3D changes to the IVDs (Fig. 3b). Virtual cross-sections through the centra revealed distinct mineral distribution patterns among the groups studied (Fig. 3b). Quantification of % mineralization robustness, here defined as the relative amount of high grayscale values in a sample (Fig. 3b, red color), showed a tendency towards lower mineralization robustness in aged spines and a significant reduction in *sp7*^*−/−*^ vertebrae (1 y) when compared with young wt vertebrae (1 y) (*P* = 0.007 9) (Fig. 3c). While wt and *sp7*^*−/−*^ vertebrae (1 y) displayed coefficients of variation (CVs) within samples of 4% and 5.8%, respectively, the aged group showed a CV of 11.95%, indicating mineral distribution heterogeneity. Mineral distribution and bone mechanical properties also depend on the organization of collagen, the most abundant component of the extracellular matrix. To examine collagen organization, we performed picrosirius red staining on paraffin sections and quantified collagen fiber thickness (Fig. 3d, e). Aged samples failed to maintain collagen organization; the matrix in aged samples showed a relative increase in thinner fibers in comparison to 1-year-old wt fish, similar to the immature collagen organization seen in 3-month-old wt fish (Fig. 3d, e). *sp7*^*−/−*^ fish (1 y) displayed a relative reduction in thick collagen fibers (*P* = 0.000 4) but no significant quantitative changes in thinner fibers, suggesting that these fish failed to develop normal bone structure and composition (Fig. 3d, e). Therefore, heterogeneous mineral distribution accompanied by deterioration of collagen organization within the bone matrix in aged samples suggests that bone quality is subtly impaired prior to the changes observed in BMD.

### Characterization of the 3D osteocyte lacunar profile in zebrafish bones demonstrates the mechanosensitivity regulatory role of *sp7*

**Fig. 4:**
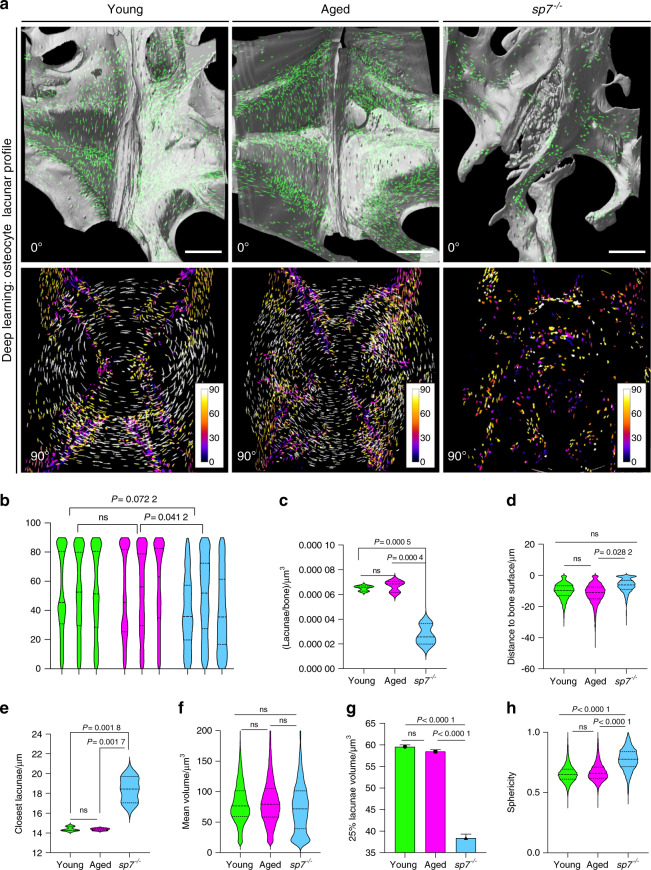
The osteocyte lacunar profile is unchanged in aged fishbut is dramatically compromised in young *sp7*^−/−^ fish. **a** The top panel presents 3D volumetric rendering and postimaging analysis of young (1 y wt), aged (3 y wt), and *sp7*^*−/−*^ (1 y) (lateral view of the IVD, 0°), showing vertebral bone and segmented lacunae (labeled in green), resulting from automated image segmentation using deep learning. Note the distribution of lacunae at the endplates and along the bone, with a dramatic reduction in *sp7*^*−/−*^. The bottom panel shows segmented lacunae (90° clockwise rotation from the lateral view of the IVD), color coded to show orientation (angle), with the center of the vertebral centrum as reference for orientation. Scale bars = 50 μm. **b** Violin plot of lacunae orientation from the center of the centrum. Data are individual zebrafish (*n* = 3). Nested one-way ANOVA, post hoc Tukey’s multiple comparisons test. *P* values are indicated. ns nonsignificant. **c** Violin plot of number of lacunae per volume of bone. One-way ANOVA, post hoc Tukey’s multiple comparisons test. *P* values are indicated. *ns* nonsignificant. **d** Violin plot of lacunae distances from the bone surface. Nested one-way ANOVA, post hoc Tukey’s multiple comparisons test. *P* values are indicated. ns nonsignificant. **e** Violin plot of the distance to the closest lacunae. Data are the mean (*n* = 3). Nested one-way ANOVA, post hoc Tukey’s multiple comparisons test. *P* values are indicated. ns nonsignificant. **f** Violin plot of lacunae volume. Note the subpopulation of lacunae showing a small volume in *sp7*^*−/−*^ zebrafish. Data are the mean (*n* = 3). Nested one-way ANOVA, post hoc Tukey’s multiple comparisons test. *P* values are indicated. ns nonsignificant. **g** The top 25% smallest lacunae volume from young (1 y), aged (3 y), and *sp7*^*−/−*^ (1 y) zebrafish were compared. One-way ANOVA, post hoc Tukey’s multiple comparisons test. *P* values are indicated. ns nonsignificant. **h** Violin plot of lacunae sphericity (circularity). Data are the mean (*n* = 3). Nested one-way ANOVA, post hoc Tukey’s multiple comparisons test. *P* values are indicated. ns nonsignificant

BMD changes could alter bone mechanical loading and influence IVDD. Osteocytes are the most abundant bone cells, strategically positioned within the bone matrix in cavities called lacunae, interconnected through cell extensions housed in small canals and canaliculi. This communication network allows osteocytes to sense mechanical loading of the bone matrix^55,56^. Variation in the 3D parameters of lacuna morphology mirror osteocyte mechanosensitivity, observed in aging human populations^57^ and in bone conditions, such as osteoporosis^55^. Osteocytes detect microfractures and initiate targeted bone remodeling but also participate in bone remodeling in the bone surface by signaling to osteoblasts and osteoclasts^58^. We explored whether variations in osteocyte lacunae morphology would be observed in aged samples and whether this observation could help to improve the present understanding of the bone mechanical properties of *sp7*^*−/−*^. We used deep learning to automate the 3D analysis of osteocyte lacunae from the SRCT image dataset (Supplementary Fig. 7A), enabling rapid and efficient retrieval of lacunae number/bone volume, shortest distance from the lacuna to the bone surface and from one lacuna to the proximal lacuna, lacunae volume, orientation and sphericity (Supplementary Fig. 7B). Lacunae showed an arrangement with a specific orientation (Fig. 4a), similar to that reported for wt zebrafish^59^. This arrangement was lost in *sp7*^*−/−*^ (1 y) (Fig. 4b). No significant differences were observed in cells/bone volume during aging (*P* = 0.925 7). However, *sp7*^*−/−*^ fish showed a dramatic reduction in cells per μm^3^ of bone (*P* = 0.000 5) (Fig. 4c), and also showed an increase in the shortest average distance between lacunae (*P* = 0.001 8) (Fig. 4e). As bone is formed, osteocytes are trapped in the newer bone matrix, more distant from the bone surface. Shallower lacunae were found in *sp7*^*−/−*^ fish (*P* = 0.028 2) (Fig. 4d). While no significant changes in the mean volume of the lacunae were identified during aging, our results showed a subpopulation of very small cells in *sp7*^*−/−*^ fish (~20 μm^3^), which was significantly different when evaluating the average volume of lacunae (Fig. 4f, g). We calculated the sphericity of the lacuna shape (perfect circle = 1), highlighting the higher sphericity in *sp7*^*−/−*^ fish compared to wt fish (*P* < 0.000 1) (Fig. 4h). While the reduced cellularity observed in *sp7*^*−/−*^ fish fits with the function of the gene in osteoblast to osteocyte differentiation^44^, we also demonstrated the role of *sp7* in regulation of the 3D profile of lacunar organization within the bone. This is suggestive of an impaired ability to sense mechanical load to regulate bone homeostasis. Interestingly, aged bone did not display significant changes in the lacunar profile, suggesting that IVDD in aging samples is independent of the lacunar profile.

### Zebrafish IVDD is characterized by the accumulation of scar tissue, dehydration and defective cellular organization

**Fig. 5:**
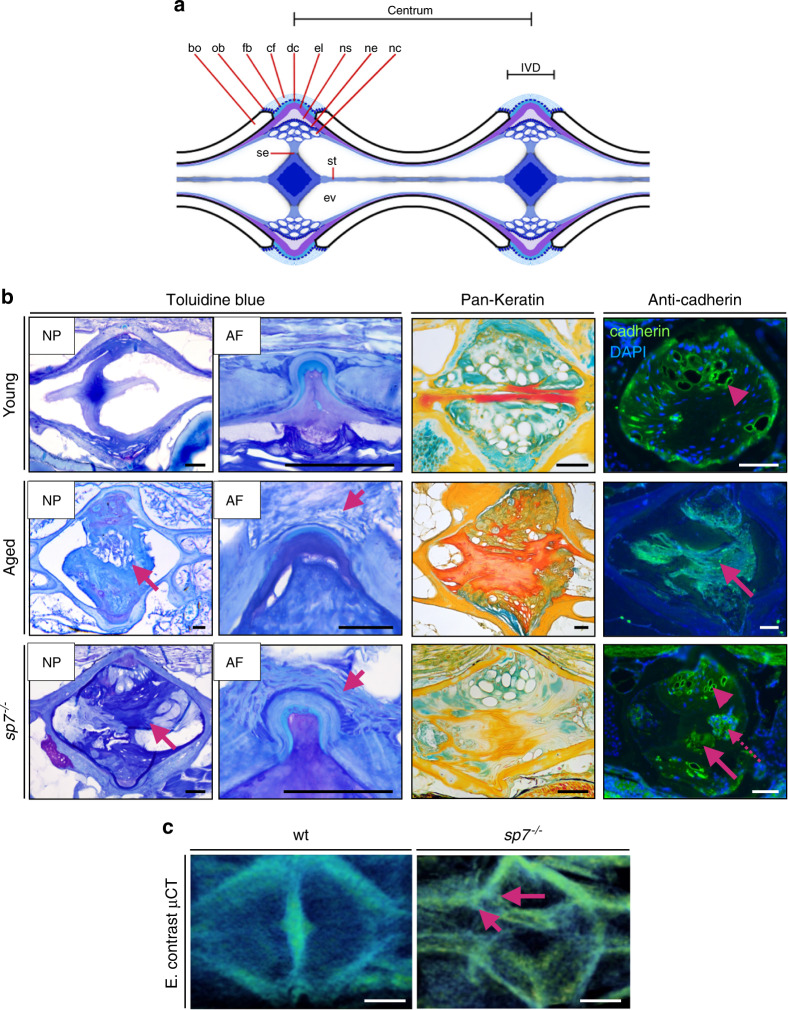
IVDD histopathology underlying 3D disc changes in zebrafish. **a** Schematic of the vertebral segments of the zebrafish. IVDD histopathology underlying 3D disc changes in zebrafish. The centrum and IVD regions are annotated. bo, bone; cf, collagen type I fiber bundles; dc, dense collagen type I matrix; el, elastin; ev, extracellular vacuole; fb, fibroblasts; nc, notochord cells, vacuolated cells and notochord epithelial cells attached to the notochord sheath; ne, notochord epithelium; ns, notochord sheath; ob, osteoblasts; se, septum; st, notochord strand. **b** Toluidine blue, pankeratin and pan cadherin immunostaining of young (1 y), aged (3 y), and *sp7*^*−/−*^ (1 y) discs. Toluidine blue of a middle section of the disc showing the NP in young, aged, and *sp7*^*−/−*^ discs (*n* = 3 per group, serial sections were analyzed). Note the fibrous and disorganized NP in aged and *sp7*^*−/−*^ (magenta arrows) discs. Higher magnification, with a focus on the AF, is shown on the right. Note collagen bundle fibers disrupted in the aged AF and loose organization in the *sp7*^*−/−*^ disc (magenta arrows). Pankeratin showing bone (yellow), keratin (orange), and glycosaminoglycans (green). Note the accumulation of keratin in aged discs and dehydration (weak stain for glycosaminoglycans) in aged and *sp7*^*−/−*^ discs. Immunostaining for pan cadherin showing the loss of cellularity in aged discs (arrow) and disorganized NPs in *sp7*^*−/−*^ discs, with vacuolated cells (pink arrowhead), acellular regions (pink arrow), and cellular agglomerates (pink dashed arrow) (*n* = 3 per group, ≥3 sections analyzed). Scale bars = 50 μm. **c** 3D volumetric rendering from enhanced (E) contrast μCT images of wt (1 y) and *sp7*^*−/−*^ (1 y) zebrafish. Note fibrous tissue organization in *sp7*^*−/−*^ zebrafish. Arrows point to an abnormal notochord sheath layer. Scale bars = 50 μm

Having characterized the changes in bone morphology occurring during IVDD, we next wanted to study cellular changes in the discs (NP and AF). For this, we performed further histological analysis of young (1 y), aged (3 y), and *sp7*^*−/−*^ (1 y) zebrafish. Overall IVD morphology was assessed by toluidine blue staining of sequential sagittal sections throughout the spine. The NP of aged samples exhibited accumulation of fibrotic tissue (scarring) and disorganized cells (vacuolated and nonvacuolated), similar features to those observed in *sp7*^*−/−*^ fish (Fig. 5b). To observe the 3D organization of the fibrotic tissue, we used phosphomolybdic acid as a contrast agent for μCT image analysis (5 μm resolution). We confirmed the 3D attachment of the fibrotic tissue with the bony walls and notochord sheath region, which suggests an involvement of the notochord sheath layer during the process of degeneration (Fig. 5c). As the main components of the notochord string are keratin and collagen type I (Supplementary Fig. 8A), we performed pankeratin staining, which labels keratin (red/orange), collagen (including collagen type I, yellow), and glycosaminoglycans (blue) (Fig. 5b). In discs from young zebrafish, we observed keratin (red) in the notochord strand (middle section). Keratin was evident throughout the scar tissue of aged NPs but not in young *sp7*^*−/−*^ fish. Similar to degeneration in mammals, dehydration of the NP was detected in *sp7*^*−/−*^ zebrafish and dramatically in aged zebrafish (pankeratin, blue). To check whether dehydration led to collapse of the vacuolated notochord cells and cell death, we used antibody staining for cadherin in the NP vacuolated cells. Interestingly, the dramatically dehydrated discs in aged zebrafish showed decreased cellularity and a lack of vacuolated cells, while *sp7*^*−/−*^ zebrafish (1 y) displayed focal areas of collapsed NP vacuolated cells with altered cellularity; in other areas, the NP vacuolated cells in *sp7*^*−/−*^ zebrafish appeared normal (Fig. 5b). These results indicate that NPs in aged samples are mostly fibrous, while in *sp7*^*−/−*^ samples, premature degeneration is observed and accompanied by cellular changes. The AF showed stretched and disordered collagen fibers in aged samples and, remarkably, in *sp7*^*−/−*^ fish (Fig. 5b, toluidine blue). However, the AF maintained the same layers of type I and II collagens and elastin. Given the extreme phenotype of *sp7*^*−/−*^, we tested whether the NP and AF would reflect degeneration or developmental abnormalities in these fish. We analyzed the expression of *sp7* using whole-mount in situ hybridization for *sp7* and imaged dissected spines of the *sp7* reporter line *Tg(Ola. Sp7:nlsGFP)*^*zf132*^. While osteoblasts at the endplates were positive for *sp7*, we did not detect *sp7* expression in the NP (Supplementary Fig. 8B, C). We analyzed histological sections of *sp7*^*−/−*^ zebrafish aged from 1 to 3 months old. At 1-month-old, despite the thin vertebral bones, we did not identify changes in the AF or NP. At 3 months old, disorganized AF and small IVD calcification were already observed (Supplementary 8C). Thus, we confirmed that changes in the vertebral column in *sp7*^*−/−*^ fish are not developmental but are a consequence of premature degeneration. We next tested the composition of the mineralized IVD by staining cryosections of wt and *sp7*^*−/−*^ zebrafish to detect calcium phosphate. We confirmed that its composition is similar to hydroxyapatite (Ca_5_(PO_4_)_3_(OH)) (Supplementary Fig. 8C). In conclusion, aged zebrafish develop IVDD in which histopathological changes include participation of the notochord sheath layer, leading to fibrotic, dehydrated, and acellular NPs—resembling those seen in human IVDD patients (collagen type I accumulation and keratin)—and destabilization of collagen fibers of the AF. Moreover, *sp7*^*−/−*^ fish develop premature IVDD with histopathological similarity to aged discs.

### 3D assessment of the zebrafish AF shows disrupted collagen fibers that prime disc herniation

**Fig. 6:**
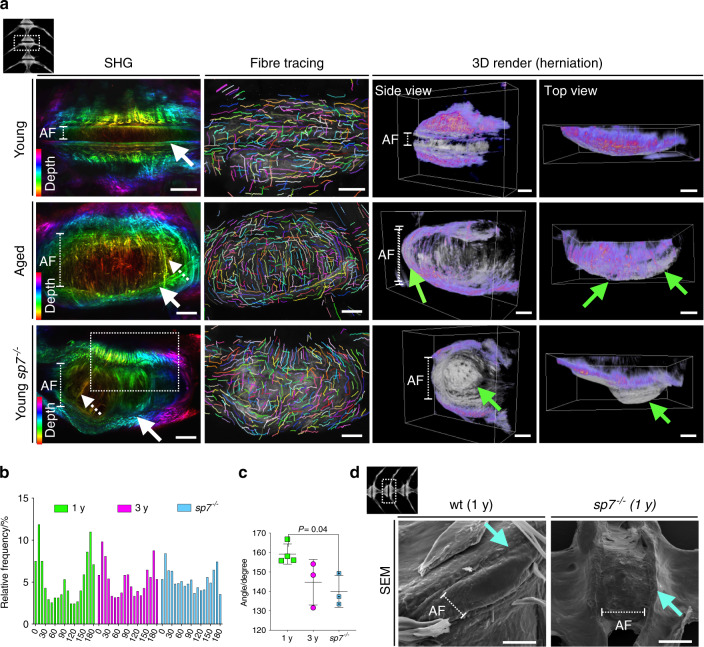
Altered collagen fiber organization and disc herniation in zebrafish IVDD. **a** Max projection of image stacks from second harmonic generation (SHG) imaging color coded by depth. Individual collagen fiber was traced using CTFire. 3D volumetric rendering from SHG images were used to visualize disc herniation in 3D using Amira (gray = back-scatter SHG; purple = forward-scatter SHG). Orientation of the vertebral column is shown at the top left of the panel. The annuls fibrosus (AF) is indicated with a dashed line. Note the variation in AF length among the groups. Smooth perpendicular fiber organization at the endplate was observed only in young samples (white arrow, SHG). Thicker fibers were observed in the AF of aged and *sp7*^*−/−*^ zebrafish (dashed arrow). Bulging discs were found in aged zebrafish and severely bulging discs were found in *sp7*^*−/−*^ zebrafish (green arrows, 3D render). Scale bars = 50 μm. **b** Relative frequency distribution (%) of angles of collagen fibers from each group. The graph was generated in Prism 8. **c** Angle of the most common collagen fibers (75% of total fibers) show abnormal orientation in *sp7*^*−/−*^ fish (*n* = 3 fish per group). One-way ANOVA, post hoc Tukey’s multiple comparisons test; data are the mean and SD. *P* values are indicated when significant (*P* < 0.05). **d** Scanning electron microscopy (SEM) of 1-year-old wt and *sp7*^*−/−*^ zebrafish (1 y). Note a smooth transition from the vertebral bone to the AF in wt zebrafish and a cliff in *sp7*^*−/−*^ zebrafish (arrow). The orientation of the vertebral column is shown on the top left. Scale bars = 50 μm

During disc degeneration, the collagen fibers of the AF are prone to rupture and tearing, leading to disc herniation, a frequent cause of back pain^5^. To investigate collagen fibers of the AF, we imaged whole IVDs in 3D through second harmonic generation (SHG) imaging using multiphoton microscopy. A healthy zebrafish disc is formed by a mesh of collagen fibers at the endplate (Fig. 6a, arrow); perpendicular fibers mark a boundary between the endplate and the AF (Fig. 6a, arrow), formed by smooth organized collagen fibers. Aged zebrafish, in contrast, have a heterogeneous mix of thick and thin fibers in the bone. Remarkably, in aged and *sp7*^*−/−*^ fish, perpendicular AF fibers were not observed, and the boundary AF/endplate appeared to be lost; thick fibers traveled through the AF (Fig. 6a, dashed arrow). We traced individual fibers using CTFire^60^ (Fig. 6a, second column: fiber tracing) and detected a significant change in the frequency distribution of the angle of fibers in *sp7*^*−/−*^ zebrafish when analyzing the whole IVD (*P* = 0.04) (Fig. 6b, c). We then explored whether collagen changes would lead to rupture of the AF and disc herniation. 3D volumetric renders showed swelling across the AF discs of aged zebrafish and localized bulging in *sp7*^*−/−*^ zebrafish (Fig. 6, green arrows), suggesting that changes in the orientation of the collagen fibers could prime herniation. Given the specific bulging phenotype of *sp7*^*−/−*^ fish and the differences in collagen fiber orientation, we performed scanning electron microscopy (SEM) of the IVDs of *sp7*^*−/−*^ and wt (1 year) fish to observe the AF in detail. Mutants showed more widespread IVD, with AF stretching, bumpy surfaces, and abrupt endplate edges (Fig. 6d). In conclusion, we showed that altered collagen fiber orientation primed bulging discs as part of the complex 3D modifications involved in IVDD in aged zebrafish and in low-BMD osteoporotic fish, demonstrating that low BMD per se does not act as a protective factor in IVDD.

### Young zebrafish displaying high bone density show an association with IVDD, suggesting a U-shaped model to explain the association between BMD and IVDD

**Fig. 7:**
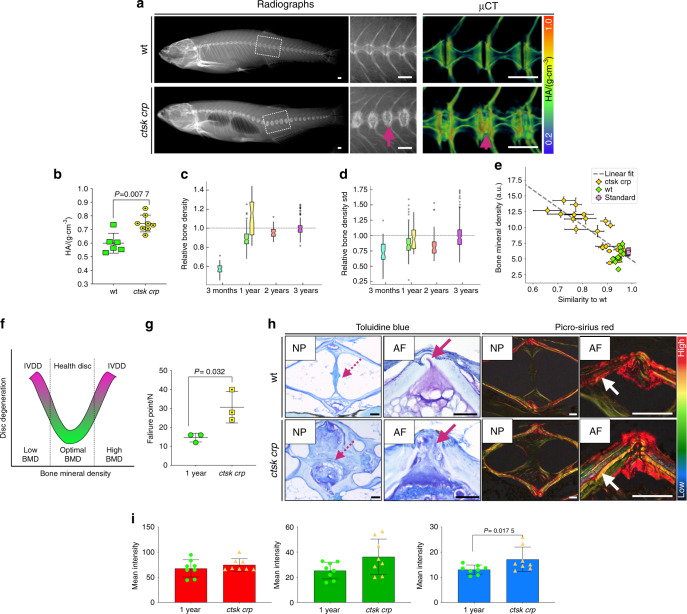
Increased bone density in *cathepsin K crp* accompanied by premature IVDD. **A** Radiographs and 3D volumetric rendering from μCT images of 1-year-old wt zebrafish and 1-year-old *ctsk crps*, color coded for bone density (TMD). Note the dramatic IVD calcification in *ctsk crps* (arrow). Scale bars = 500 μm. **B** TMD showing differential density in *ctsk crps* [wt *n* = 6 (3 males, 3 females), *ctsk crps* *n* = 8 (4 males, 4 females)]. Standard fish lengths: wt = 3.5 (0.3 SD); *ctsk crp* = 3.5 cm (0.09 SD). The graph was generated in Prism 8. Nonparametric, two-tailed, Mann–Whitney test; data are the mean and SD. *P* values are indicated. **C** Relative bone density from the vertebral centra in aging fish. The notch plot was scaled by the average value from the 3-year-old centra (cyan = 3 months wt; green = 1-year old wt; yellow = 1-year-old *ctsk crps;* orange = 2-year-old wt; magenta = 3-year-old wt). The graph was generated in Python. **D** Within-sample standard deviation if bone density. The notch plot was scaled by its average value from the 3-year-old centra (cyan = 3-month-old wt; green = 1-year-old wt; yellow = 1-year-old *ctsk crp;* orange = 2-year-old wt; magenta = 3-year-old wt). The graph was generated in Python. **E** Cross-correlation between mineral density and wt morphology of the discs was calculated for wt, *ctsk crp* and wt standard samples. Pearson correlation coefficient = −0.85, *P* = 7.8e^−14^. The graph was generated in Python. **F** Proposed U-shaped model to describe the association between BMD and IVDD. Either an abnormal increase or decrease in BMD is linked to IVDD. **G** Failure point during vertebral compression. Nonparametric, two-tailed, *t*-tests; data are the mean and SD. *P* values are indicated. The graph was generated in Prism 8. **H** Histological sections of wt and *ctsk crp* (1 year) zebrafish stained with toluidine blue and picrosirius red, with a focus on the NP and AF regions. Higher magnification of the AF from another histological section is shown on the right. Disorganized NP (dashed arrows, toluidine blue) and abnormal AF, displaying loss of the elastin layer (arrows, toluidine blue), were detected in *the* affected IVD of c*tsk crps*. Picrosirius red staining shows different colors in the bone of *ctsk crps* (higher magnification), indicating bone quality impairment (white arrow). Scale bars = 50 μm. **I** Quantification of the thickness of collagen fibers from picrosirius red staining (blue = thin, green = medium, red = thick) (1-year-old *n* = 8, *ctsk crp* *n* = 8). Parametric, two-tailed, *T*-test; data are the mean and SD. *P* values are indicated. The graph was generated in Prism 8

As increased vertebral body BMD was linked to disc degeneration^23^, we aimed to investigate young fish exhibiting high BMD. Cathepsin K is a protease expressed by osteoclasts and is important for bone resorption. Mutations in *CTSK* cause pycnodysostosis, a disease characterized by osteosclerosis (bone hardening and high BMD)^61^. *CTSK* is associated with estimated BMD and lumbar spine area (mskkp.org). Furthermore, genes associated with osteoclast function are associated with BMD and changes in aging^47^. CRISPR technology in zebrafish has been shown to be highly efficient, such that G0 skeletons display phenotypes consistent with those of homozygous mutants^62^. We used CRISPR/Cas9 to generate *ctsk* mosaics (crispants, *crps*) showing high BMD throughout the vertebral column. We analyzed 1-year-old *ctsk crp* by radiographs (*n* = 25), μCt (*n* = 8), and histological sections (*n* = 3). We detected severe endplate sclerosis, osteophytosis and IVD calcification in 70% of injected fish, mild in 15% of injected fish and no IVD changes in 15% of injected fish. By calculating centra TMD in *ctsk crps*, we confirmed the significant increase in TMD in affected vertebrae (sclerosis) of crispants (G0s), from 0.6 to 0.75 g·cm^−^^3^ HA (*P* = 0.007 7) (Fig. 7). Automated centra segmentation in comparison to aging wt showed the highest density in *ctsk crps* (28 centra), with a standard deviation similar to that of 3-year-old wt fish (Fig. 7c, d). We used radiographs to calculate the correlation between X-ray attenuation (pixel intensity, representative of BMD) and morphology of the vertebral column at the discs (wt = 13, wt standard (the same reference wt fish added to all X-rays) = 9, *ctsk crp* = 25). Remarkably, higher bone mineral densities were systematically associated with IVD deformities (Pearson correlation coefficient = −0.85, *P* = 7.8e^−14^) (Fig. 7e). An inverse correlation was observed between density and morphology; thus, higher density was positively associated with abnormal disc morphology, suggesting a U-shaped model linking BMD and IVDD (Fig. 7f). *Ctsk crps* subjected to vertebral compression displayed significant resistance to the applied force, with failure points at 30 N (*P* = 0.032) (Fig. 7g) and reduced Young’s modulus (elasticity) (Supplementary Fig. 9A). When we sectioned *ctsk crps*, we revealed IVDD, fibrous NP and disrupted AF (Supplementary Fig. 9B), and the lack of the elastin layer in discs exhibiting abnormal morphology (Fig. 7h). Picrosirius red staining revealed a spectrum of colors associated with an increase in thinner fibers in *ctsk crps* (*P* = 0.017 5) (Fig. 7i), signifying altered bone quality through changes in collagen fiber organization. By analyzing young fish with high BMD, we detected endplate deformities and disc calcification associated with IVDD. Therefore, our data suggest that the association between BMD and IVDD best fits a U-shaped model in which both increased and decreased BMD can exacerbate IVDD (Fig. 7g).

## Discussion

IVDD is the most common cause of back pain, a leading cause of disability worldwide and a global concern as the population ages^1,3^. Understanding the relationship between IVDD and aging-related degenerative diseases would support drug repurposing and the development of less invasive therapeutics. Here, we showed evidence of osteoporosis in aged zebrafish. We characterized zebrafish IVDD in 3D and shed light on the relationship between low BMD and IVDD and between high BMD and IVDD. We provided evidence that disproved osteoporosis is a protective factor in IVDD and showed a positive correlation between high BMD and IVDD. Our findings suggest a U-shaped model to explain the relationship between BMD and IVDD. Genetic factors regulating bone formation, resorption and bone quality are candidates for disc disease and potential targets for therapeutics.

We characterized zebrafish disc degeneration in 3D, focusing on bone, the NP, and the AF. This resulted in a more holistic appreciation of phenotypes not often analyzed together, highlighting the potential of zebrafish as a spontaneous model to recapitulate human IVDD. Aged zebrafish displayed bone morphological abnormalities comparable to those of mammals: bone sclerosis, osteophytosis, disc narrowing, misalignments and disc calcification. Cellular transformations within the NP were followed by 3D modifications in the architecture of the discs, with fibrosis and disc herniation, recapitulating chronic cases of human IVDD. Several animal models and mechanisms to replicate IVDD have been proposed, but due to IVDD complexity, all forms of replication have limitations. The sand rat and the chondrodystrophoid dog are the most studied species in which spontaneous IVDD occurs^63^. The mechanical load of discs in quadrupedal mammals differs from that in humans, with gravitational loading occurring perpendicular to the vertebral axis. As zebrafish swim through a viscous medium, the distribution of load in the zebrafish spine is axial, similar to bipeds, including humans^39^. The loss of notochordal cells in humans is associated with NP degeneration and IVDD^64^. Zebrafish have persistent notochordal cells in the IVD^65^, which can be disadvantageous when modeling human IVDD; however, persistent notochordal cells are common in most animal models of IVDD^63^. Interestingly, we demonstrated IVDD to be accompanied by the loss of notochordal cells and fibrosis, similar to the outcomes in humans, suggesting an important role of vacuolated cells in preventing degeneration. The ability to generate mutants in a genetically tractable zebrafish model can enable the identification of key molecular players controlling cellular modification within the NP during aging, which can in turn help to disentangle potential genetic factors of IVDD. Another limitation of our model is the lack of cartilaginous endplates. Whereas the endplates in mammals are formed by a layer of cartilage under different differentiated statuses, the bony endplates in zebrafish are in direct contact with the collagenous notochord sheath layer; thus, the translational interpretation from phenotypic assessment of the region to humans is not direct and should be considered.

Calcification of IVDs is part of the degeneration phenotype of IVD. Calcification has been reported in 60% of elderly human cadavers^66^ and is also detected in dogs, sheep and mice^67^. Recent studies have demonstrated that disc calcification is dependent on genetic background^67^. Our results indicate that calcification is also part of zebrafish IVDD and associated with genes involved in bone formation and remodeling, as we detected calcification in nearly 80% of aged spines and in younger zebrafish showing misalignments; severe calcification was observed in zebrafish carrying mutations in *sp7* and *cathepsin K*. Disc calcification is observed in other zebrafish mutants linked to collagenopathies, such as *col1a1* ^+/−^^29^, *col1a1b*^*−/−*^^29^, *bmp1a*^*−/−*^^29^, *crtap*^*−/−*^^30^, *p3h1*^*−/−*^^30^, and *plod2*^*−/−*^^62^. Human conditions affecting bone quality, such as osteopenia, rickets, pseudogout disease, and juvenile chronic arthritis, also support the association of abnormal collagen maintenance and bone quality with IVDD^68^. Recently, the association of disc calcification with cell death, matrix remodeling, changes in calcium/phosphate homeostasis and cell transformation has been reported^67^, similar to the changes that we detected in zebrafish IVDD. Therefore, a common mechanism of disc calcification might prevail among different species. Interestingly, the zebrafish notochord sheath layer mineralizes as part of the normal vertebral centrum^69^, a process that is *sp7* independent but linked to the expression of *entpd5* and the retinoic acid signaling pathway^70,71^, suggesting *entpd5* and retinoid acid as potential genetic candidates involved in disc calcification.

Bone fragility is the predominant phenotype in osteoporosis, and the risk of fracture is assessed through BMD. For the first time, we described bone density heterogeneity and uneven spatial distribution in the zebrafish spines during aging, with the increased density mostly limited to the endplates—the regions of highest morphological variation. Zebrafish continue growing throughout their life course, and bone is formed in areas of osteoblast activity^26^. The increased density at the endplates can be explained by continuous bone deposition by osteoblasts followed by bone deformation due to mechanical load changes. Bone deformation during degeneration led to local increases in BMD in aging zebrafish, a fact also observed in degenerative lumbar spines of elderly women, whose deformations of the spine coincided with elevated BMD; consequently, a large portion of those with osteoporosis was not detected^43^. We showed other parameters demonstrating bone quality deterioration in aged zebrafish in parallel with an increased risk for fractures, indicating osteoporotic bone in aged zebrafish. BMD heterogeneity and spatial distribution have also been associated with vertebral fracture in human studies^72^. Previous measurements of vertebral regional BMD in human participants from the Framingham Heart study showed that the BMD decrease in aging is spatially nonuniform (decrease in the central region and increase in the vertebral edges), as in zebrafish, and associated with disc health^24^. Measurements of bone density in zebrafish using μCT imaging might not detect microstructural BMD differences during aging^36,37^. Current μCT limitations on detecting subtle amounts of minerals in normal zebrafish bones, exemplified by the nondetection of already mineralized bones in larvae and juveniles, mean that results using this method should be viewed with caution. By improving image resolution, we were able to detect a trend towards reduced BMD (TMD), suggesting the presence of osteoporotic bone.

The controversial relationship between osteoporosis and IVDD has long been an area of debate^13,17–19,73^. We have demonstrated premature IVDD both in aged wt fish and in low-BMD *sp7*^*−/−*^ fish, contradicting the possible benefit of prolonged osteoporosis in preventing IVDD. This finding is also supported by experiments inducing osteopenia through movement restriction in zebrafish showing disc calcification, which suggests IVDD^74^. By genetically manipulating *cathepsin K*, we suppressed bone resorption, showing an inverse correlation between bone density and IVDD, supporting an association between high density and IVDD^14,23,75–79^. Alterations in mechanical loading and bone remodeling could lead to shape modifications, nonuniform BMD, and IVDD^24^. We also analyzed the organization of mechano-sensing cells and osteocytes. While changes in osteocyte lacunae morphology and distribution, such as reduced lacunae volume and a trend towards a more spherical shape with increasing age^57^, have been reported during the aging process in mammalian studies, we did not detect significant changes in aged zebrafish vertebrae. However, empty lacunae could not be evaluated with our analysis, and osteocyte death^55^, a feature in mammalian aged bone, cannot be ruled out. Bone heterogeneity in aged samples cannot be explained by osteocyte lacunar organization from our experiments, suggesting changes to osteocyte activity rather than their number and organization per se. However, we showed that the osteoblast transcription factor *sp7* regulates osteocyte number and function, which suggests that s*p7* has a role in osteocyte mechanosensitivity and bone maturation, which fits with the reduced BMD observed in mutants. From our findings, abnormal positive or negative fluctuations in BMD values increase the risks of IVDD, pointing to a U-shaped model to explain the highly controversial association between BMD and IVDD.

Our work and tools developed provide supportive evidence for the development of therapeutics that improve bone quality, could potentially ameliorate osteoporosis and could enhance IVDD treatment. Alendronate is a potent bisphosphonate and one of the front-line drugs for osteoporosis. It increases BMD, suppresses osteoclast activity and rescues bone mass and microstructure. It has been reported to retard the progression of lumbar IVDD in ovariectomized rats^18,20^. The use of the antiresorptive agent calcitonin has also been demonstrated to prevent IVDD in ovariectomized rats^21,22^. Moreover, several cathepsin K inhibitors are currently in clinical trials as potential drugs for osteoporosis and osteoarthritis^80,81^. However, we found that loss-of-function *cathepsin K* mutations accelerated IVDD, suggesting that antiresorptive drugs, if administered for an extended period of time, might lead to the progression of disc disease. Controlled exercise can induce bone formation and mineralization that could improve bone quality, as shown in controlled swimming experiments in zebrafish^39^, representing potential alternatives to ameliorate IVDD. Our results suggest a role in IVDD for genes associated with BMD involved in the regulation of osteoblast differentiation and bone resorption. Finally, functional studies in zebrafish have the potential both to test putative genetic associations with IVDD and to support testing of potential novel therapeutics for IVDD. Taken together, zebrafish could be a powerful model in the fight against the highly prevalent global health burdens represented by IVDD and back pain.

## Materials and methods

### Zebrafish husbandry and lines

Zebrafish were raised and maintained under standard conditions^82^. Animal experiments were ethically approved by the University of Bristol Animal Welfare and Ethical Review Body (AWERB) and conducted under the UK Home Office project license. *sp7*^hu2790^ mutants were previously generated as part of an ENU-induced mutation consortium^83^ and described by us^31^. The mutation leads to a premature stop codon and truncation of the protein before the three-zinc-finger DNA binding domains^31^. Osteoblast transgenic line *Tg(Ola. Sp7:nlsGFP)*^*zf132*^ (ref. ^84^) and *sp7*^hu2790^ mutants were maintained as previously described^31^.

### Cathepsin K crispant (ctsk crp)

Two gRNAs targeting exon 5 of *cathepsin K* (chr16: 29563627–29563649, chr16: 29563739–29563761) were synthesized and incubated with GeneArt Platinum Cas9 nuclease (Invitrogen) prior to injection into one cell embryo as previously described^85^. To check gRNA efficiency, DNA was extracted from 20 individual injected larvae at 2 days postfertilization (dpf) and were subsequently evaluated by PCR amplification (F: FAM-GTCAGAACCACTTTGACTTCCATTT; R: TGCTGACGTATCTGAAGGCG) and fragment length analysis (ABI 3500)^86^. gRNAs were tested and passed our 90% efficiency criteria (multiple peaks identified in >18 out of 20 larvae analyzed)^86^. The average height of the wt peaks showed a reduction of 80%, meaning that our G0s had a rate of 20%wt/80% mutant. Two-thirds of the cells are expected to carry biallelic mutations^87^. *Cathepsin K crispants* (*ctsk crps*) G0s were maintained for up to 1 year old for mosaic analysis.

### Alizarin Red S staining

Alizarin Red S staining was performed using standard protocols^88^. Images were acquired with Zeiss Stemi 305 (1.2 megapixel Wi-Fi camera). Live Alizarin Red S staining was carried out as previously described^89^.

### Microcomputed tomography

Adult fish were fixed in 4% PFA for 1 week followed by sequential dehydration in 70% ethanol. Fish heads were scanned using an XT H 225ST micro-CT scanner (Nikon) with 21 voxels sized 21 µm and 21 voxels sized 5 µm for detailed geometric analysis using an X-ray source of 130 kV, 53 µA without additional filters. Images were reconstructed using CT Pro 3D software (Nikon). Amira 6.0 was used for image analysis and to generate 3D volume and surface renders. Classification of spinal morphology was performed blinded and adapted from the Kellgren–Lawrence classification system^90^. Briefly, a severity score (from −2 to 2) was assigned to each of the samples in each of the categories analyzed: fusions, misalignments, osteophytosis, sclerosis, narrowing and calcification. For unaffected samples: score = −2, for mildly affected samples: score = −1, for mildly/moderately affected samples: score = 0, for moderately affected samples: score = 1, and finally for severely affected samples: score = 2. Numbers were exported to Prism 8, and a heat map graph was generated. For calculations of TMD, defined as a measurement restricted to within the volume of calcified bone tissue^91^, the centra were segmented, and the mean gray values were retrieved. Gray values were calibrated with phantoms of known densities (0.25 and 0.75 g cm^3^ of CaHA) and used for density calculations, as previously described^92^. For soft tissue µCT, samples were incubated with contrast-enhanced solution, phosphomolybdic acid (0.2%), for 20 days prior to µCT. Amira (6.0) was used to acquire pictures of the samples.

### Spinal segmentation for relative bone density calculation

The 3D images were first downsampled by a factor of 0.5. An intensity threshold was applied to retain only the 0.7 percentile, which broadly corresponds to most of the bone material. A sparse matrix was then used to represent the dataset where every voxel is a point identified by a quadruplet of 3D coordinates and intensity. The head region was then automatically identified and removed based on the higher density of points. The spine was determined through a smooth B-spline with a large smoothing parameter, exploiting the transverse symmetry of the vertebrae. The spine voxels were then projected on the curvilinear spline to compute a one-dimensional density profile. The intervertebral spacings were later identified through a peak-finding algorithm and used to generate a mask for a labeling algorithm for the core of the vertebrae. The labels were finally propagated with a label-spreading algorithm employing *k*-nearest-neighbors, with *k* = 12. The numbers of specimens for each wt age range and the *ctsk crp* group were as follows: 3-month-old wt (6), 1-year-old wt (37), 2-year-old wt (10), 3-year-old wt (27), *ctsk crp* (6).

### Radiographs

Adult fish (1 y, *n* = 35; 2 y, *n* = 25; 3 y, *n* = 70) were radiographed using a MultiFocus digital radiography system (Faxitron), and the same settings were used for all the images: 45 kV, 5 s of exposure and 0.46 mA. Phantoms of known densities (0.25 and 0.75 g cm^3^ of CaHA) were added to each X-ray picture for gray value calibrations. Live fish were anesthetized prior to imaging.

### Synchrotron radiation-based μCT (SRCT)

Young, aged and *sp7*^*−/−*^ (*n* = 3 for each group) fish were scanned at the TOMCAT beamline of the Swiss Light Source (SLS) with an isotropic voxel size of 0.33–0.68 μm, 1 501 projections, 120–180 ms exposure time and 14 keV X-ray energy at a sample-to-detector distance of 20 mm. Reconstructions were processed using single propagation distance phase retrieval with a *β* value of 3.49 × 10^−8^ and a *δ* value of 1.66 × 10^−6^. 3D models were generated from reconstructions using automated segmentation tools in Amira 6.0.

### Percentage of mineralization SRCT (mineralization robustness)

Cross-sectional pictures were taken at the middle plane of each SRCT centrum using the orthoslice function in Amira (6.0). Three orthoslice pictures (*n* = 3 for each group) scanned under the same threshold parameters were taken per sample. Postprocessing was carried out in Fiji/ImageJ^93^. Images were binarized (under the same parameters), and four regions of interest (ROIs) were selected following the four vertices of the centrum. Measurements of % area were calculated (number of black pixels/total pixel area, here referred to as % mineralization).

### Deep learning for 3D analyses of osteocyte lacunae

Training and application of the U-Net model^94^ for segmentation and morphological analysis of the zebrafish spine and embedded cells was achieved using five discrete steps. Following the creation of the U-Net model (steps 1–3), new image stacks could be fully processed using steps 4 and 5 only. Step 1: pixel classification models were generated for a subset of the image stacks to eventually be processed. For each stack, approximately 12 cropped 2D images (412 × 690 px^2^) were extracted and used to train pixel classification models using the WEKA Trainable Segmentation plugin for ImageJ/Fiji^93,95,96^. The identified classes were “background” (nonbone/cell regions), “edge” (boundary between “background” and “bone”), “bone”, “cell” and “cracks” (fractures in the background region; optional, depending on image stack). Step 2: the models were applied to all slices of their corresponding image stacks to generate probability maps for the identified classes (to improve computational efficiency, stacks were cropped in the *XY* plane to remove excess background regions). Application of the WEKA classifiers and subsequent generation of U-Net training data used the MIA workflow automation plugin for ImageJ/Fiji^97^. The process of generating U-Net training data involved binarizing the relevant probability maps and identifying objects (bone, cells and cracks) as contiguous regions of foreground-labeled pixels^98^. Detected objects were passed through appropriate size filters to remove instances where noise had been spuriously identified as objects. To remove noncell objects accidentally detected in the dark halo, often present at the bone-background interface, any identified cell objects with centroids outside the bone were discarded. Class-labeled images were created (0: background, 1: bone, 2: edges, 3: cell, and 4: cracks) and, along with the corresponding raw images, were reduced to overlapping 512 × 512 px^2^ tiles; these tiles were saved to a folder structure compatible with the data loader for U-Net training. Step 3: raw and class-labeled image pairs were used to train a Keras U-Net model^99,100^. The model was trained using a batch size of 16, with data augmentation enabled, and a workstation with dual 10-core Intel Xeon CPUs, 128 GB RAM and Nvidia Titan XP GPU (CUDA enabled) was used. Step 4: the trained U-Net model was used to predict pixel class labels for full image stacks. Step 5: segmentation of bone and cells from U-Net probability maps followed a similar logic to that used in Step 2. Probability maps were binarized, and objects were detected using connected component labeling before being passed through size filters. The volumes of all cell and bone objects were measured, as well as the nearest neighbor (centroid-to-centroid) distance between cells. Additionally, cell sphericities and orientations were calculated by fitting ellipsoids to each object^101^.

### Morphometric analysis

2D outlines of each centrum were prepared (Adobe Illustrator) (*n* = 8 for each group) and were converted into 200 *XY* coordinates with a common origin located at the anterior tip using TpsDig 2.17 (ref. ^102^). Coordinates were converted to sine and cosine components using Hangle Fourier transformations^103^ and superimposed using Procrustes superimposition in Past 2.17 (ref. ^104^). To qualitatively assess morphospace occupation and shape variation, the data were subjected to principal component analysis between groups and built in Past (PCA, Past 2.17). Quantitative differences were assessed using one-way nonparametric MANOVA (Past 2.17). Shape deformities (displacement of landmarks from the mean shape in the direction of principal components) were plotted using eigenvalue scales, scores of −2 and +2, and displayed with hot and cold color schemes.

### Histology

Fish were decalcified in 1 mol·L^−1^ EDTA solution for 20 d hickness throughout the entire vertebral column. Slides were dewaxed and stained sequentially every third slide with toluidine blue^51^ and every other slide with picrosirius red^36^. Images were acquired on a Leica DMI600 inverted microscope using ×20 and ×40 oil objectives, and LAS software; a DFC420C color camera was also used for toluidine blue stained sections. A polarizing filter was used for pictures of the picrosirius red stained sections. *sp7*^*−/−*^ fish at 1 month, 6 months, and 1 year of age were sectioned (5 µm) using a Prosan HM360 microtome after embedding in Technovit. Observations were performed using a Zeiss Axio Imager Z1 microscope with a 5 megapixel CCD camera.

### Immunohistochemistry

Immunostaining was performed on histological sections. Anti-collagen I (1:200, Abcam, ab23730), anti-collagen II (1:500, DSHB), and anti-pan cadherin (1:500, Abcam, ab6529) were used as primary antibodies, and DyLight 488 goat anti-mouse IgG and DyLight 550 goat anti-rabbit IgG (1:1 000, Molecular Probes) were used as secondary antibodies. Briefly, sections were dewaxed and washed several times with PBST. We used proteinase K solution (10 µg·mL^−1^) as a retrieval agent and incubated sections for 30 min at 37 °C in this solution. Sections were blocked in 10% BSA for 1 h before incubation with primary antibody overnight at 4 °C. Sections were washed several times with PBST and incubated with secondary antibodies for 4 h at room temperature. Sections were counterstained with DAPI (1:5 000 in PBST) to reveal nuclei. Images were taken using a Leica DMI6000 inverted epifluorescence microscope using ×20 dry lenses and a Leica DFC365FX camera.

### Confocal imaging

Three-month-old *Tg(Ola. Sp7:nlsGFP)*^*zf132*^ wt fish were live stained with Alizarin Red for 15 min, transferred into a freshwater fish system, and cultured in MS222 after 1 h. Dissected spines were mounted in 1% low-melting point agarose and imaged using an SP5 Leica confocal microscope using LAS software.

### SHG imaging for collagen fiber quantification

Dissected spines were mounted in 1% low-melting point agarose (*n* = 3 for each group). SHG images were acquired using a ×25/0.95 water dipping lens, 880 nm laser excitation, and simultaneous forward and backward detection (440/20) with a Leica SP8 AOBS confocal laser scanning microscope attached to a Leica DM6000 upright epifluorescence microscope with multiphoton lasers allowing fluorescent acquisition and SHG of the same sample and *z*-stack acquisition. Microscope parameters for SHG were set as previously described^105^. Maximum projection pictures were assembled using Fiji^106^ and color coded by depth. CtFire was used for analysis of fiber orientation^60^.

### Scanning electron microscopy

One representative fish spine (≥4 IVDs) from each group (young wt and *sp7*^*−/−*^) was processed for SEM. Fish were dehydrated with 99% ethanol and mechanically dissected, during which as much of the muscle and soft tissue surrounding the spine was removed as possible. Dissected spines were dehydrated using a Leica CPD300 Critical Point Dryer, mounted on SEM stubs and sputter coated with gold/palladium before being imaged in an FEI Quanta 200 FEGSEM (Thermo Fisher Scientific).

### Spinal compression

Motion segments consisting of three posterior vertebrae and accompanying soft tissue were dissected for each specimen (*n* = 3 for each group), and the resistance of these segments to compressive force was studied using a custom designed MTS in the Bruker SKYSCAN 1272 micro-CT system following a recently described method^42^. Each motion segment was antero-posteriorly compressed in the MTS at an increasing series of compressive forces between 1–20 N. Once each 1 N incremental increase in force was attained and stabilized in the MTS, a micro-CT scan was performed at 60 KeV X-ray energy, 50 W current, and 5 µm isotropic voxel size using a 0.25 mm aluminum filter. A total of 1 501 projections were collected with 400 ms exposure time over a 180° rotation. Reconstructions of scans (performed using filtered back projection in NRecon v. 1.7.1.0) were analyzed in ImageJ/Fiji^106^ for failure patterns in bone or connective tissues. Vertebral compression graphs were created by fitting a Gaussian distribution to averaged strain versus stress data values for each subsample using the “fit” function in MATLAB (R2019a; The MathWorks, Inc., Natick, MA, USA).

### Density and disc morphology cross-correlation calculation

Radiograph files were converted to RGB, and overall intensity was rescaled according to the intensity of phantoms (scanned concurrently). The intensity profile of the vertebral column was measured by drawing a line through 7 vertebrae in the transitional region of the vertebral column (40 pixel width). The standard intensity profile of wt zebrafish was calculated from the same wt fish added to each X-ray. Morphological similarity was measured from the peak value of the cross-correlation between the measured vertebral intensity of *ctsk crispants* and the wt standard profile (*ctsk crp* *n* = 25, standard *n* = 9, and wt *n* = 13). The cross-correlation values were rescaled by the maximum value of the autocorrelation of the standard profile. Therefore, the maximum value of the cross-correlation corresponds to 1 (perfect cross-correlation). The density (BMD) was taken as the integration of the measured intensity profile divided by the length of the measured vertebral line (7 vertebrae in the transitional vertebral region). The background was subtracted when calculating the BMD. The background was defined as a linear fit of the local minima. The Pearson correlation coefficient was calculated for statistical analysis. All the analysis was performed using Python.

### Human *SP7* patient evaluation

Evaluation of the patient was conducted under ethical consent and at the Limb Malformations & Skeletal Dysplasia Clinic, NRC, Egypt.

### Statistics

Graphs and statistical analyses were generated by GraphPad Prism, version 8, and Python. The results are expressed as the mean ± SD. Information on the statistics used is provided for each graph in the figure legends. Fish length and sex were matched when comparing groups, and information on fish length and sex is provided in the figure legends.

## Supplementary information

Supplementary Figures

## Data Availability

Data are available at the University of Bristol data repository, data.bris, at 10.5523/bris.3g29uwsxfme722nt1dhii6nol9.
