# Supplementary material for: 3D assessment of intervertebral disc degeneration in zebrafish identifies changes in bone density that prime disc disease
Source: Bone Res. 2021 Aug 31;9:39. doi: 10.1038/s41413-021-00156-y (PMC8408153; doi:10.1038/s41413-021-00156-y)
Supplement: Supplementary file 1 — Supplementary Figures [file 41413_2021_156_MOESM1_ESM.pdf]

3D assessment of intervertebral disc degeneration in zebrafish identifies changes to bone density that prime disc disease

Authors: Erika Kague[1]*, Francesco Turci[2], Elis Newman[1], Yushi Yang[2, 3, 4], Kate Robson Brown[5,6], Mona S. Aglan[7], Ghada A. Otaify[7], Samia A. Temtamy[7], Victor L. Ruiz-Perez[8], Stephen Cross[9], C Patrick Royall[2,10], P Eckhard Witten[11] and Chrissy L Hammond[1]*

Supporting information listing

Item	Type	Tittle
Supplementary Fig. 1	Figure	IVD abnormalities detected through radiographs and Alizarin Red staining.
Supplementary Fig. 2	Figure	Shape deformities at the endplates during ageing.
Supplementary Fig. 3	Figure	Workflow for automated zebrafish vertebral centra segmentation.
Supplementary Fig. 4	Figure	Types of measurements retrieved from vertebral centra segmentation of young and aged zebrafish.
Supplementary Fig. 5	Figure	One year *sp7-/-* show lower bone density and dramatic abnormalities in the IVD.
Supplementary Fig. 6	Figure	Radiographs of the trunk of a 15 years old patient carrying a homozygous truncation in *SP7* (c.1052delA).
Supplementary Fig. 7	Figure	Workflow for segmentation and analyses of bone and osteocytes lacunae from SRCT images using a single U-Net model.
Supplementary Fig. 8	Figure	IVD changes in *sp7-/-* are part of premature degeneration.
Supplementary Fig. 9	Figure	Fibrotic and disorganised IVD characterize IVDD in *ctsk crp* and reduced bone elasticity modulus.

Supplementary Figures and legends

Supplementary Figure 1

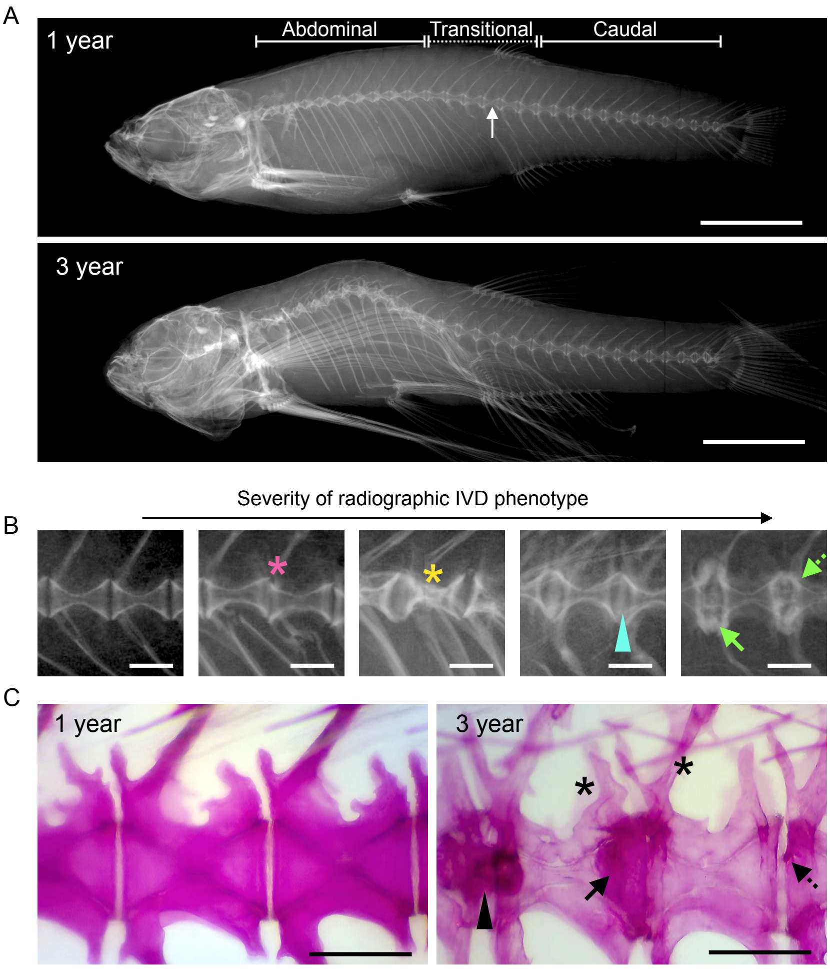

Supplementary Fig 1. IVD abnormalities detected through radiographs and Alizarin Red S staining.

A) Radiographs of 1 and 3 year wt. The three areas of the axial skeleton are shown (abdominal, transitional and caudal), in accordance with (De Clercq, Perrott et al. 2017). Transitional vertebrae were chosen for TMD calculation from μCT data. Note spinal curvature in aged sample. Scale bars: 3 mm.

B) Examples of compromised radiographic IVDs, from normal (extreme left) to severely affected (far most right). Misalignment (magenta asterisk), compromised centrum shape and borders (yellow asterisk), IVD calcification (cyan arrowhead), sclerosis (green dashed arrow) and fusions (green arrow). Scale bars: 500 μm,

C) Alizarin Red S staining of 1 and 3 years old spines. Aged spines show uneven and lighter centra staining, osteophytes (dashed arrow), sclerosis (arrow), IVD calcification (arrowhead) and shape changes of arches and spinous processes (asterisks). Scale bars: 500 μm.

Supplementary Figure 2

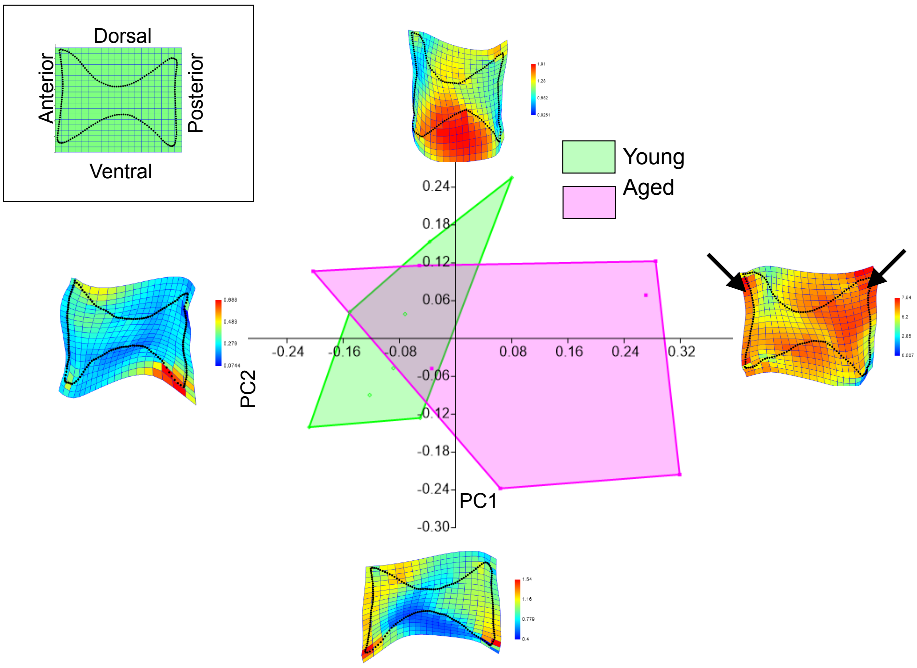

Supplementary Fig. 2. Shape deformities at the endplates during ageing.

Morphometric analysis showed distinct shape of vertebral centrum when comparing young and aged samples (n= 8 for each group). Principal Component (PC) and PC2 displayed. Shape deformation of the vertebral centrum, displayed with 20 grids colour-coded for points of stress (deformation) in accord to the standard deviation of each PC. Note that in aged samples the deformation of the centra is located towards the endplates (arrows, red colour grids). Top left, vertebral centrum without any deformation, orientation is indicated. Non-parametric, ANOVA, Bonferroni-corrected p-values. Euclidean distance measured. Graph generated in Past2.17.

Supplementary Figure 3

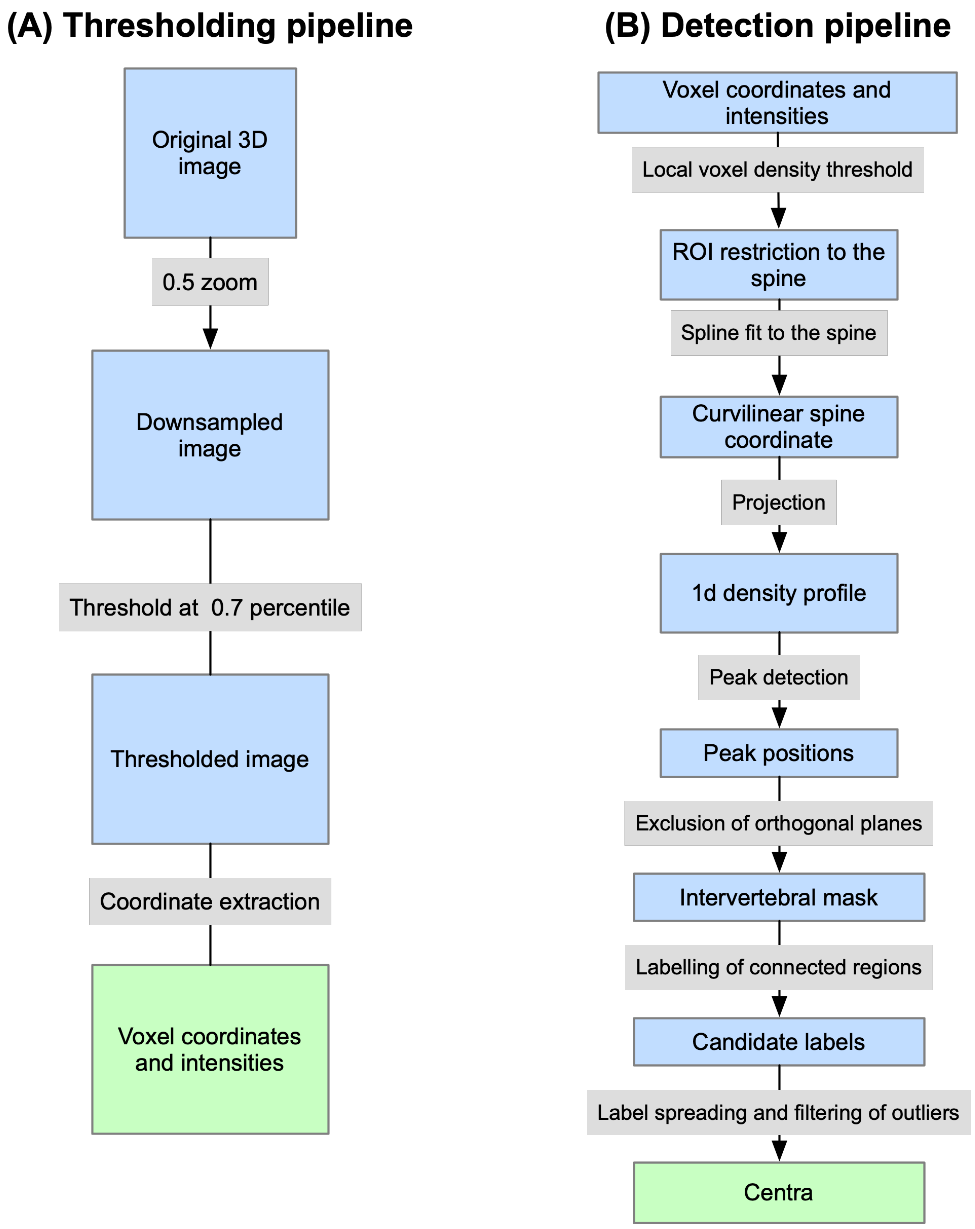

Supplementary Fig. 3. Workflow for automated zebrafish vertebral centra segmentation. A) Pipeline for the first step of automation of centra segmentation, focused on thresholding the image. To reduce computational costs, images are downsampled, and only the 0.7 highest intensity percentile is retained. The thresholded image is then transformed into a set of coordinates and intensities.

B) Pipeline for the second step of automation of centra segmentation, focused on detection of vertebral centra (vertebrae). To detect the vertebrae we first automatically detect the region of interest (ROI) excluding head and fins. We then focus on the spine region and establish a reference curvilinear coordinate system over which we project the positions of the spine voxels. This one-dimensional profile is used to identify intervertebral separations and a mask for the labelling of connected regions. Through label spreading we recover the centra.

Supplementary Figure 4

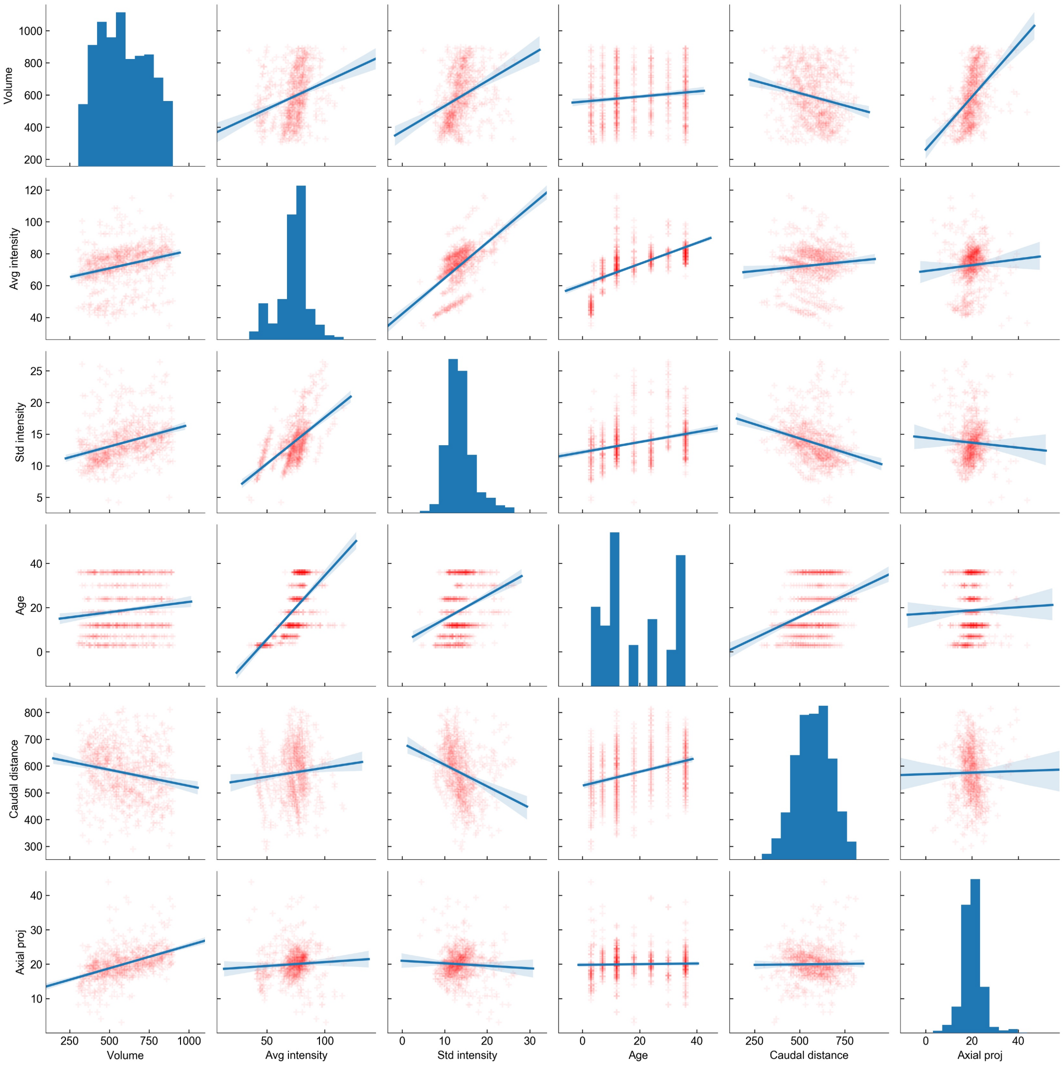

Supplementary Fig. 4. Measurements retrieved from vertebral centra segmentation of young and aged zebrafish.

Graphs showing age (wt, in months) of volume, average intensity (bone density), standard (std) deviation of intensity per vertebra, caudal distance (distance to the caudal fin), axial projection (length). Each point represents one centrum. Taking ageing as a comparison, small increase in volume, average intensity, std intensity, and length is observed in wt spines. Higher densities are observed in centra farther from the tail. Fish numbers: 1y (n= 36), 2y (n= 16) and 3y (n= 34). Graphs generated in Python.

Supplementary Figure 5

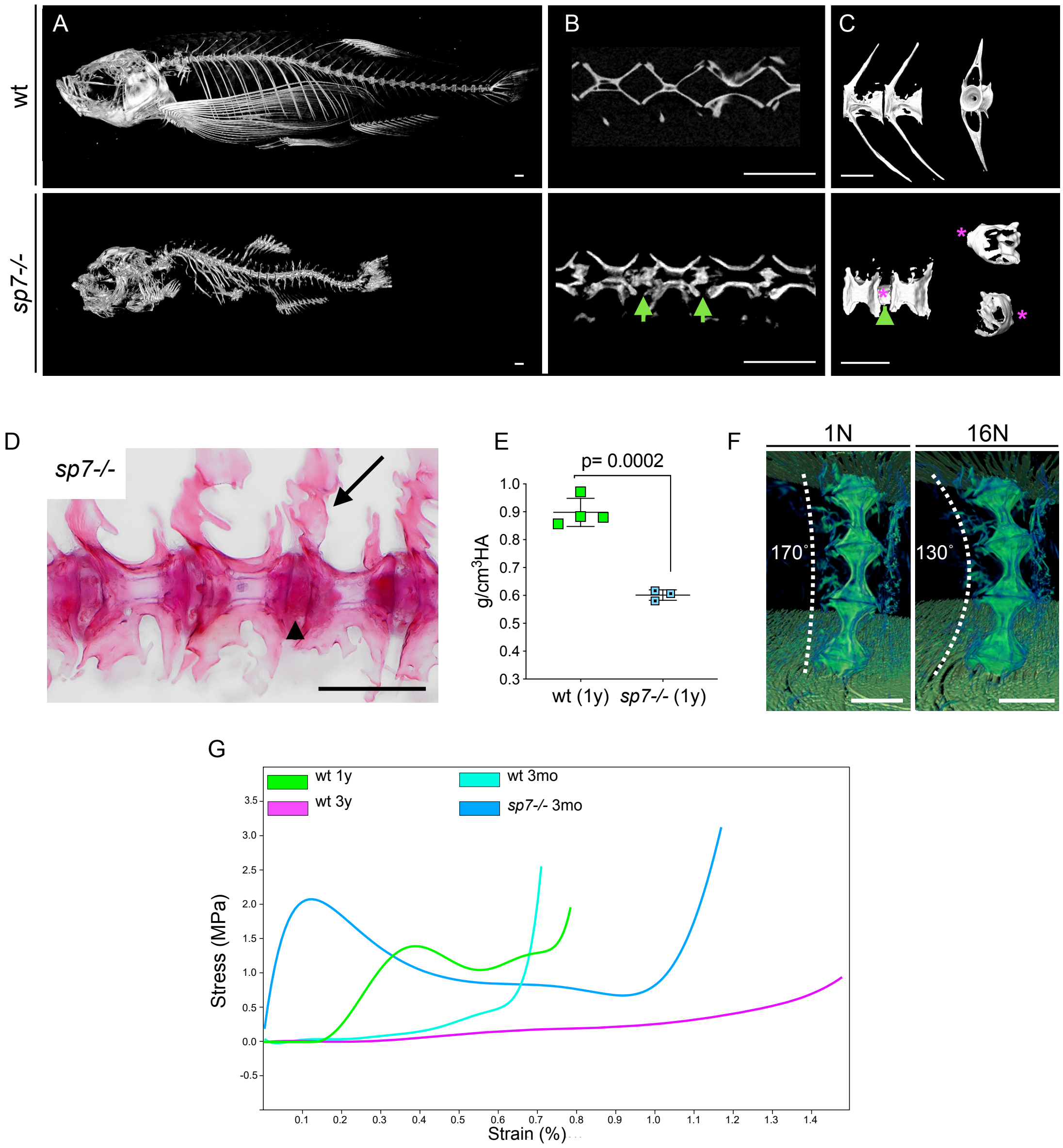

Supplementary Fig. 5. One year *sp7-/-* show lower bone density and dramatic abnormalities in the IVD.

A) 3D volumetric render from µCT of 1y old wt and *sp7-/-* 1y. Scale bars= 500 µm.

B) Sagittal orthoslice across the spine. Arrows point to IVD calcification in *sp7-/-*. Scale bars= 500 µm.

C) Segmentation of two consecutive vertebrae from wt 1y and *sp7-/- 1y*. Note abnormal shape in vertebrae centra in *sp7-/-*. IVD calcification was segmented (asterisk and arrowhead), revealing not a solid mineralized rod, but calcified sheet layers. Asterisks positioned at the same region to help with orientation of IVD calcification. Scale bars not placed for segmented IVD calcified rods. Scale bars= 500 µm.

D) Alizarin Red showing a region of the *sp7-/-* spine. Note abnormal shape of centra and arches (arrow) and IVD calcification (arrowhead). Scale bars= 500 µm.

E) TMD values of 1 year wt and sp7-/-, calculated from µCT data acquired under the same conditions. Non-parametric, two-tail, Mann-Whitney test; data are mean SD. P-values are indicated.

F) 3D volumetric render of the same *sp7-/-* sample during the compression (1N and 16N). Note the bent spine at 16N. Scale bars= 500 µm.

G) Stress x Strain from the vertebral compression experiment to show Young's modulus. Lines represent average of three samples. Graph generated in MatLab.

89 **Supplementary Figure 6**

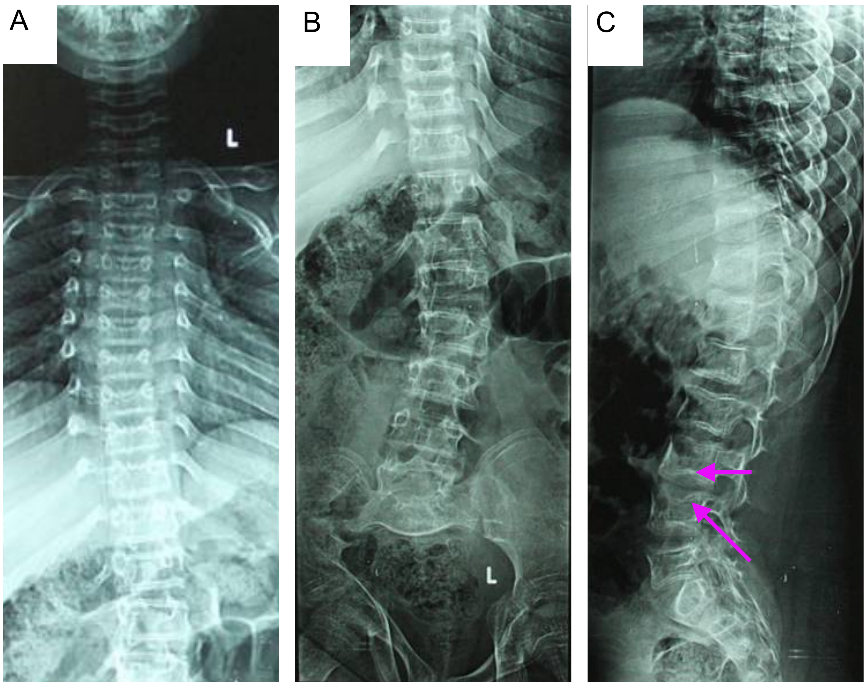

90

Supplementary Fig. 6. Radiographs of the trunk of a 15 years old patient carrying a homozygous truncation in *SP7* (c.1052delA).

A and B) Anterior posterior x-ray view of dorsolumbar spine showing mild platyspondyly and scoliosis with mild deviation to the right in the thoracic region and convex to the left in the lumbar region. Posterior aspects of ribs are deformed with smooth kinking.

C) X-ray lateral view of dorsolumbar spine showing generalised osteopenia, biconcave thoracic and lumbar vertebrae with irregular transverse lines of sclerosis at the upper and lower vertebral cortical endplates and increased intervertebral disc space (arrows).

Supplementary Figure 7

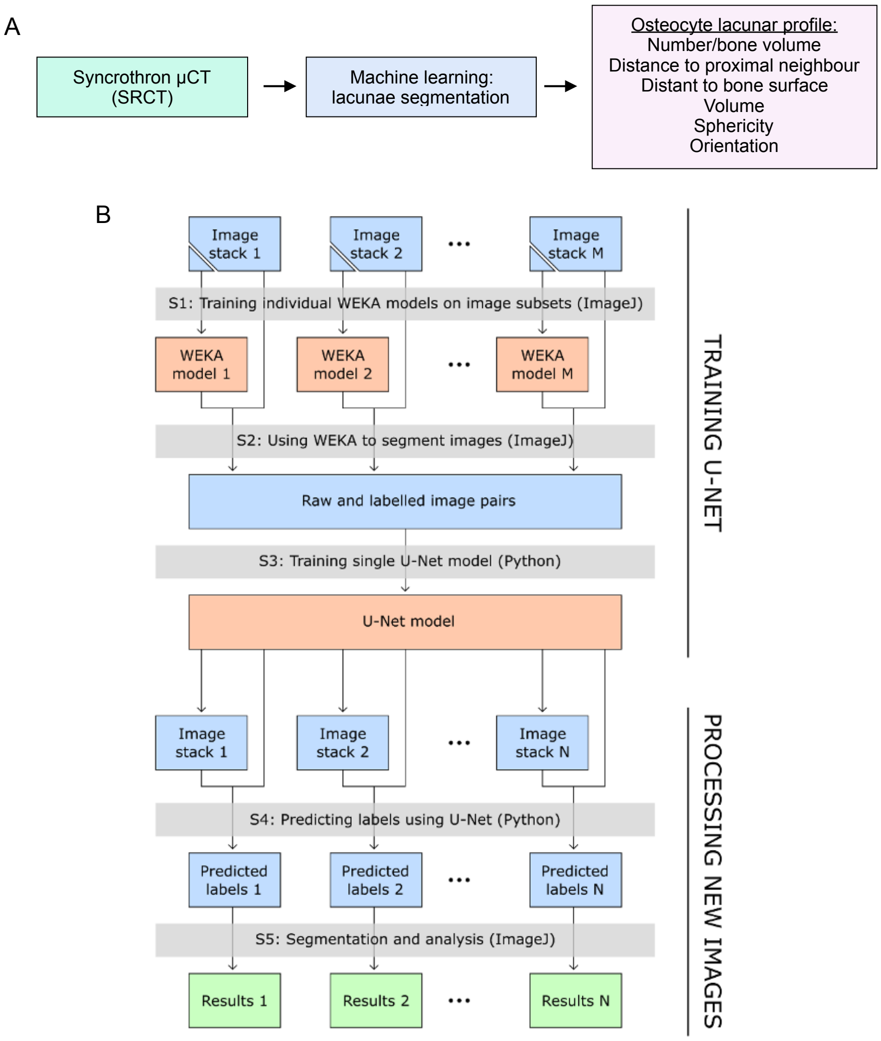

Supplementary Fig. 7. Workflow for segmentation and analyses of bone and osteocytes lacunae from SRCT images using a single U-Net model.

A) Machine learning was used to retrieve osteocyte lacunae information from SRCT data. B) The workflow was split into two parts: (1) Creation of the single U-Net model for pixel classification and (2) use of the U-Net model on previously-unseen images to facilitate downstream segmentation and analysis. The two parts of the workflow were split into five discrete steps (labelled S1-S5) and use either Python or ImageJ/Fiji. S1: Example 2D regions from a subset (*M*) of the images to be ultimately processed (*N*; where $M \leq N$) were extracted and used to train WEKA pixel classification models (one classifier created per image stack). S2: The WEKA classifiers were used to create probability maps for all slices in the corresponding example image stacks. These probability maps were binarised and converted to pairs of raw and class-labelled images using a MIA workflow (v0.14.13). The image pairs were then converted to 512x512 px^2 overlapping tiles and combined into a single dataset. S3: A single Tensorflow/Keras U-Net model was trained on the tiled image pairs, which can subsequently be applied to all SRCT images acquired in a similar manner. S4: The U-Net model was applied to image stacks (both previously seen and unseen) and outputs probability maps for classes of interest. S5: Probability maps were segmented and analysed using MIA to yield measurements of lacunae counts, morphology and bone morphology.

121 **Supplementary Figure 8**

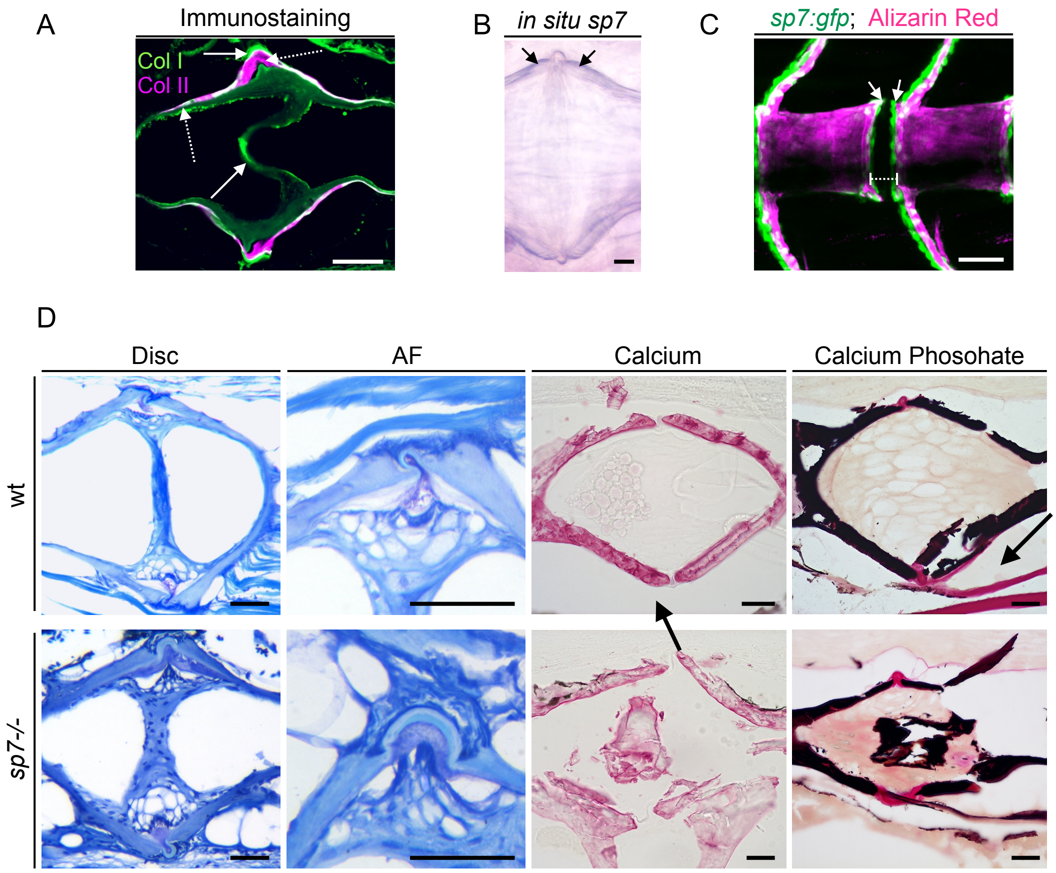

122

123

Supplementary Fig. 8. IVD changes in *sp7-/-* are part of premature degeneration.

A) Histological section of the intervertebral disc of wt, immune-stained for collagen type II and collagen type I. Collagen type I localises to the notochord strand and layer in AF (arrows), collagen type II in AF and notochord sheath (dashed arrows). Scale bars= 50 µm.

B) Whole- mount *in situ* hybridisation for *sp7* showing the IVD region. *sp7* is expressed at the endplates, no signal was observed in the NP. *In situ* hybridization was carried out as previously described (Kague, Roy et al. 2016). Scale bars= 50 µm.

C) Maximum projection from confocal stacks of *Tg(Ola.Sp7:nlsGFP)*^{zf132} live stained with Alizarin Red S. *Sp7*+ in osteoblasts at the endplate (arrows). IVD (dashed line). Scale bar = 50 µm.

D) Histological sections of wt and *sp7-/-* (3 months old) to show the first signs abnormalities in the AF (Toluidine Blue). Cryosections of 6 months old fish stained for Alizarin Red S to show that calcium is the component of disc calcification. Von-Kossa show calcium phosphate in calcified IVD (arrows) and bones. Scale bars= 50 µm.

Supplementary Figure 9

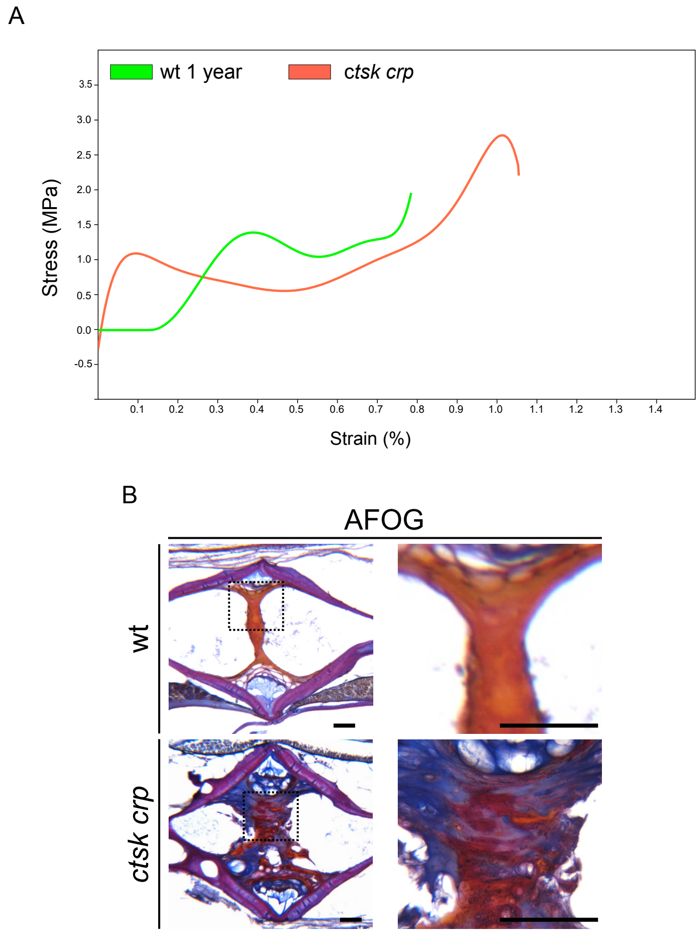

Supplementary Fig. 9. Fibrotic and disorganised IVD characterise IVDD in *ctsk crp* and reduced bone elasticity modulus.

A) Stress x Strain graph to represent Young's modulus of wt and *ctsk crp* (1 year) during vertebral compression. Lines are average of each group (n= 3). The exponential ramp of each line correspond to Young's modulus (elasticity). The peak of each curve shows the failure point. Graph generated in MatLab.

B) Histological sections of wt and *ctsk crp* (1 year) stained with AFOG (Acid Fuchsin Orange G) to show fibrosis (orange) and disorganization of the NP. The region within the dashed box is shown in higher magnification. Scale bars = 50 μm.
